# The Moderating Effect of Learning Experience on Learning Motivation and Learning Outcomes of International Students

**DOI:** 10.3389/fpsyg.2022.913982

**Published:** 2022-06-28

**Authors:** Jingxiao Zhang, Gangzhu Sun, Lin Xu, Inayat Khan, Weidong Lv, Simon P. Philbin

**Affiliations:** ^1^School of Economics and Management, Chang'an University, Xi'an, China; ^2^School of Civil Engineering, Zhengzhou University, Zhengzhou, China; ^3^School of Foreign Languages, Northwest University, Xi'an, China; ^4^School of Management, Northwest Polytechnical University, Xi'an, China; ^5^School of International Education, Chang'an University, Xi'an, China; ^6^School of Engineering, London South Bank University, London, United Kingdom

**Keywords:** international students, learning motivation, learning experience, learning outcomes, experimental study and application

## Abstract

With the increasing level of internationalization in higher education, the number of international students in mainland China is rapidly increasing. However, limited research has considered that student results may be affected by a reduced motivation to learn. Therefore, the aim of this research is to explore the effect of the learning motivation on the learning outcomes of international students and the moderating role of learning experience. A sample of 130 international students from 23 countries studying in mainland China was analyzed. The study found a significant correlation between the learning motivations and international students' learning outcomes. It was also determined that learning experience has significantly enhances the relationship between learning motivation and the learning outcomes of international students. This study contributes to the higher education literature on learning motivation by students and learning outcomes.

## Introduction

The number of international students in colleges and universities has become an important index for measuring the level of internationalization of higher education (Johnson, 2012; Urban and Palmer, 2014). In this regard, China has attracted a large number of international students from relatively underprivileged countries by means of initiating scholarships programs designed to develop Chinese universities and boost the corresponding world educational ranking (Gao and Hua, 2021). However, international students often face more challenges studying abroad, including cultural conflicts, environmental adaptation, language barriers, and other issues. International students also vary widely in their motivation to learn and their learning capabilities and especially in an unfamiliar environment in a different country, which can significantly impact achievement of the learning outcomes (Liao et al., 2012; Chang, 2014). The nature and intensity of the motivation of international students can determine the most suitable learning methods, processes, and eventually impact the academic results achieved by students and their overall performance in the academic institution. This area has attracted much research attention and the motivation of students is regarded as one of the most influential factors affecting achievement of learning outcomes by students (Liu et al., 2012; Hsieh, 2014).

On the other hand, research into learning experience of international students including how knowledge is acquired has generally focused on the study of influencing factors (Barton et al., 2015; Heng, 2018). Several recent studies agree that learning experiences are related to the expected learning outcomes (e.g., Grayson, 2008; Hill, 2013; Gao and Hua, 2021). Furthermore, there is a lack of empirical studies on the moderating effect of learning experience on achievement of learing outcomes.

Recently scholars have recognized the importance of learning motivation and learning outcomes (Drew and Watkins, 2011; Wang et al., 2012). International students in higher education are generally considered to be highly motivated (Valle et al., 2003). Recent research has mainly focused on the factors that influence the performance of students and adaptation to the learning environment when studying abroad (Luo and Jamieson-Drake, 2015; Richart, 2015), as well as the learning motivation of international students in different countries (Petzold and Peter, 2015; Zhou, 2015). However, learning motivation and learning experience have been studied in relative isolation, with the impact of both on learning outcomes being assumed as independent. The present study attempts to explore the interrelationships involved between these constructs (i.e., learning motivation and experience with learning outcomes).

According to the latest data from the Chinese Ministry of Education, China is now the largest Asian destination for studying abroad (MOE, 2019). In 2018, the number of international students from Asian and African countries in China was 376,605 (76.52% of the total foreign students in mainland China), with an increase of 0.62% over last year. Currently there are 495,185 international students persuing studies in China (Gao and Hua, 2021). The Chinese government has set up a series of scholarship programs and offered a huge number of low cost or free tuition places for international students coming from Africa, Pakistan and other nearby countries. This has raised the requirements and challenges for international student education. It can be observed that researchers are gradually shifting their attention toward these issues too (Ding et al., 2010; Yu, 2010). However, to the best of our knowledge, there has been very little reserach on the learning motivation of international students and the learning experience in China.

To address this gap, this study develops a conceptual model to investigate the effects of learning motivation and learning experience on the learning outcomes of international students in mainland China. The study also investigates the moderating effect of the learning experience on the relationship between learning motivation and learning outcomes. The findings of this study improve our understanding of the learning motivation of international students helps to promote the development of such educational activities in mainland China.

## Theoretical Background

### Effect of Learning Motivation on Learning Outcomes

The study by Kraiger et al. (1993) provided a systematic framework for the measurement of learning outcomes. Learning outcomes are considered the consequence of a complex system involving the interaction of student personality traits with learning processes (Kumpas, 2012). Indeed, learning outcomes are considered as one of the most important research topics in higher education (Ruban and McCoach, 2005). In this context, learning motivation is recognized as one of the most important factors influencing the learning process (Minnaert and Janssen, 2011).

Learning motivation promotes the achievement of students' learning outcomes of students. This is also true of international students (Bailey and Phillips, 2015; Chue and Nie, 2016). The study by Deci and Ryan (1985) on self-determination theory identified that individuals tend to pursue achievement of their basic psychological needs of competency, self-determination and belonging. This theory holds that when students are intrinsically motivated, their behavior is the consequence of their interest in the activity itself as well as their personal beliefs (Deci and Ryan, 2000). Both aspects have been researched and have been found to significantly and positively predict learning performance (Zhu and Leung, 2011). Motivation can be divided into intrinsic and extrinsic motivation (Deci and Ryan, 2000), and further subdivided according to the degree of individual autonomy in behavior. Intrinsic motivation is divided into intrinsic motivation for knowledge, achievement and stimulation. Whereas, extrinsic motivation includes identified regulation, introjected regulation, and external regulation. External regulation focuses on external conditions, while introjected regulation and identified regulation internalize the activity outcomes. These two latter regulations contain more self-determination aspects (Chirkov et al., 2007). A learning requirement could (theoretically and positively) affect the students' sense of self-regulation and identification by making students internalize the extrinsic motivators that push them to learn.

In recent decades, the learning experience of international students has gained the attention of many scholars (Boonen et al., 2021; Zhao et al., 2021; Tian et al., 2022). International students initially choose to study abroad to increase their academic knowledge, promote personal development, widen career prospects and enhanced potential economic benefits (Schaub and Tokar, 2005; Eder et al., 2010; Wang and Brown, 2014; Chue and Nie, 2016). Intrinsic motivation is a highly autonomous type of motivation and embodies the concept of self-determination. In this regard, some scholars have highlighted the importance of appealing to the intrinsic motivations of international students (Hill, 2013; Richart, 2015), and especially if the academic institutions want to host the international students for longer periods and achieve positive learning outcomes (Chirkov et al., 2008).

Different background characteristics and learning motivation effectively predict different learning outcomes in higher education (Hsieh, 2014). In self-determination theory, motivation to *knowledge* relates to acquiring new knowledge, exploring the world and satisfying individual curiosity (Vallerand, 1997). Under the motivation to *achieve*, students can achieve inner satisfaction by succeeding in academic tasks (Vansteenkiste et al., 2010; Gaudreau, 2012). Although Martínez et al. (2011) found that achievement motivation had no direct impact on cognitive outcomes in their study of Spanish college students.

*Experience stimulation* is the most autonomous form of internal motivation in self-determination theory (Deci and Ryan, 2000), whereindividuals engage in certain activities for the inherent pleasure. As a consequence, pleasure can stimulate the motivation of students to participate in deeper learning. This, in turn, allows students to secure improved cognitive learning outcomes (Wang et al., 2000; Drew and Watkins, 2011; Lin et al., 2017). In this regard, Chirkov et al. (2007) found that Chinese students studying abroad who were intrinsically motivated adapted better than those stimulated by expectations or the external environment.

*External regulation* of self-determination theory does not relate to the perception of the activity itself, but to the external requirements that avoid punishment or penalties. This regulation is enforced with the expectation that individual behavior will change to avoid the negative outcomes of inadequate behavior. Wentzel (2010) found that social competence, including social responsibility, social status, and self-regulation process are significantly correlated with learning outcomes.

*Identified regulation* is the most autonomous external motivator in self-determination theory (Deci and Ryan, 2000). It means that individuals consider their behavior as important and relevant to value; that is, identified regulation involves the internalization of external activity outcomes. Wu and Tai (2016) found that perceived value and success expectations are both positively correlated with learning outcomes.

Although many research studies have been conducted into the learning motivation of international students in China (Boonen et al., 2021; Hua and Gao, 2021; Li et al., 2021; Song and Xia, 2021; Zhao et al., 2021; Tian et al., 2022). These studies have mainly focused on the interculture learning (Song and Xia, 2021; Zhao et al., 2021), language learning (Zhu et al., 2021), and personal networks (Li et al., 2021). However, there is a lack of empirical research on the types of learning motivation of international students and achievement of learning outcomes in China. To fill this gap in the extant literature, this study analyzed the attitude of international students in China and based on self-determination theory divides learning motivation into external regulation, identified regulation, achievement, knowledge, and experience stimulation. We argue that the learning motivation of international students may improve achievement of their cognitive and non-cognitive learning outcomes. The study has the intention of finding out how different types of learning motivation can play a positive role in improving learning behavior, attitude, and cognitive learning of international students in mainland China. Based on the above arguments, the following hypotheses have been synthesized from the literature:

*Hypothesis 1*: *Learning motivation has a positive influence on non-cognitive learning outcomes*.
*Hypothesis 2: Learning motivation has a positive influence on cognitive learning outcomes*.

### Effect of Learning Experience on Learning Motivation and Learning Outcomes

The experiential learning theory by Kolb and Kolb (2012) proposed that learning behavior is the process of creating knowledge through experience. Learning experience contributes to deeper student learning, improving motivation and behavior (Ruhanen, 2005). Learning experience provides international students with a rapid insight into differences across learning environments while studying abroad (Pope and Sand Peter, 2015). Current research on learning experience focuses on its connotation, measurement, and influencing factors (Thompson and Dahling, 2012). On this matter, reference information concerning the underlying mechanisms connecting learning experience, motivation, and learning outcomes is rather scarce. The moderator variable can change the intensity and direction of the relationship between these variables (Baron and Kenny, 1986), where the moderator can either increase or decrease the intensity, or change the positive and negative effects between variables.

The previous international experience of international students is an important factor in their willingness to study abroad (Petzold and Peter, 2015; Lörz et al., 2016). Existing literature on international students in China has focused on different factors, including international students' intercultural experience (Hua and Gao, 2021; Song and Xia, 2021; Zhao et al., 2021), living experience Gao and Hua, 2021), language learning experience (Hua and Gao, 2021) and personal network (Li et al., 2021). However, few scholars have investigated the learning experience of international students. It is argud that international students with learning experience may have a motivation to learn in the host environment. Learning experience may enhance the relationship between learning motivation of students and achievement of learning outcomes (both cognitive and non-cognitive). Therefore, the following hypotheses have been synthesized from the literature:

*Hypothesis 3*: *Learning experience has a positive effect on the relationship between learning motivation and non-cognitive learning outcomes*.
*Hypothesis 4*: *Learning experience has a positive effect on the relationship between learning motivation and cognitive learning outcomes*.

**Figure 1:**
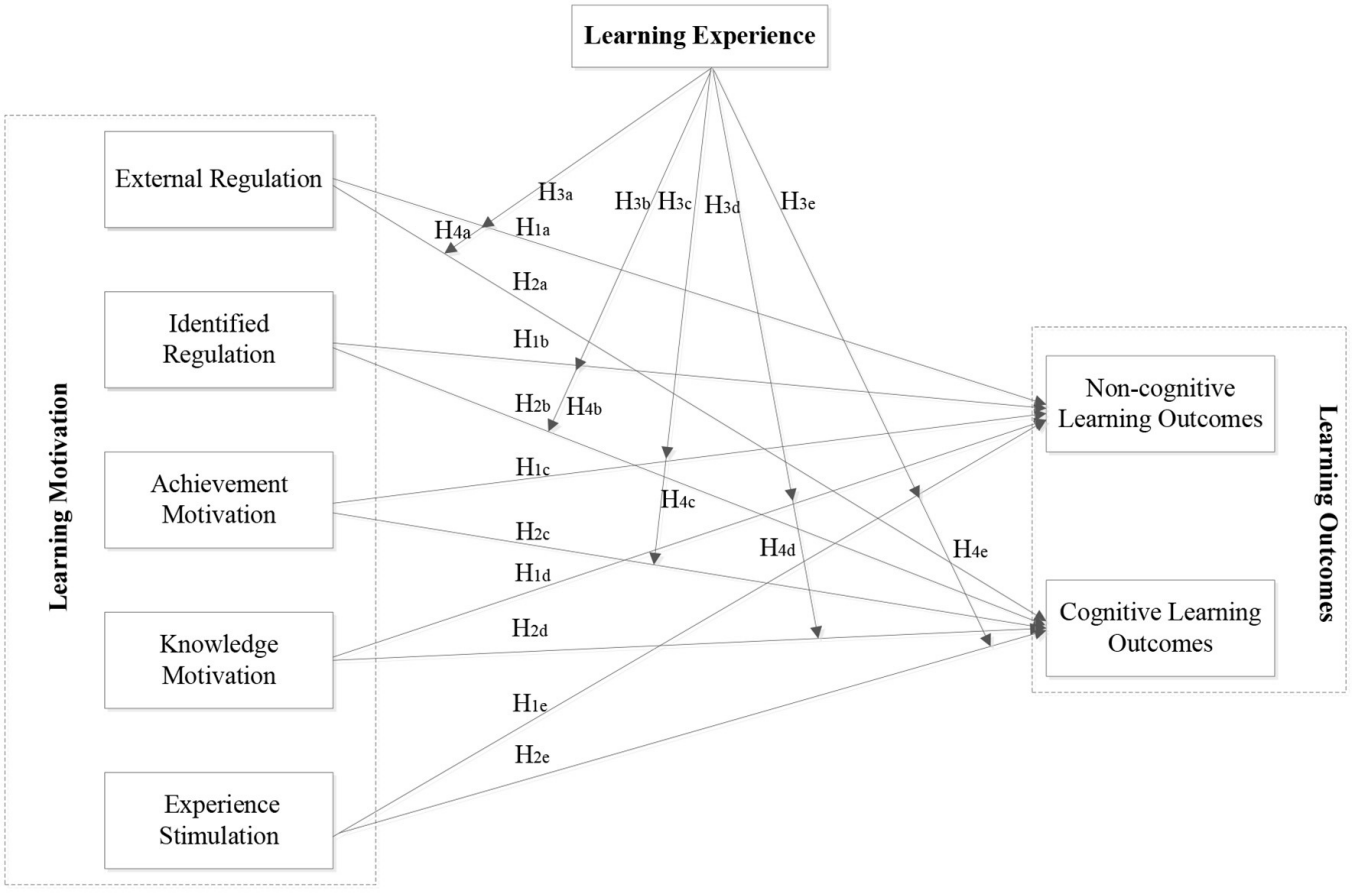
Model describing the (hypothesized) moderating influence of learning experience between learning motivation and learning outcomes.

Figure 1 illustrates the hypotheses from this study. The conceptual model depicts the (hypothesized) moderating influence of learning experience between learning motivation and learning outcomes.

## Research Methods

### Questionnaire Survey

The sample of the study was international students in different universities in Xi'an located in Shaanxi Province, China. A questionnaire survey was conducted with international students studying in mainland China in 2021 and the convenience sampling technique was used for the inqury. The first section of the questionnaire recorded the demographic information (such as gender, age, native language, etc.), along with details on the aim of the questionnaire and the research study, section contents as well as guarantees of confidentiality of the participant responses. The participants were international students in different universities in Shanxi Province, Xi'an China from 23 countries around the world. The study team published a survey announcement on the International Student Affairs official website and social media platform, and recruited 130 international students to participate in the survey. The survey was open for 3 weeks from July 9th to July 31st, 2021) and deemed valid for research purposes. Previous studies on the experience of international students in China included eight (Li et al., 2021), nine (Zhao et al., 2021), and twenty participants (Song et al., 2021). In contrast to previous studies, this study had a large number of participants (105 completed the questionnaire), which is more appropriate, reliable. This figure accounts for 10% of the total number of international students in Shaanxi Province, Xi'an China. Table 1 provides the detail of demographic information of the population sample.

**Table 1:** Summary of the international students' sample demographics.

**Statistical variables**		**Effective samples**	**Percentage**
Gender	Male	71	67.6
	Female	34	32.4
Educational background	Basic education	1	1.0
	Undergraduate	90	85.7
	Postgraduate	14	13.3
Age	17–18	2	1.9
	19–20	20	19.0
	21–24	70	66.7
	25–30	12	11.4
	31–39	1	1.0
Language	Chinese	11	10.5
	English	92	87.6
	Arabic	1	1.0
	French	1	1.0
Sponsor	Oneself	3	2.9
	Parents	84	80.0
	Relative	3	2.9
	University	9	8.6
	CSC	6	5.7

### Semi-structured Interview

Semi-structured interviews provide more open discussion and an improved understanding of the research, where certain adjustments can be made to questions based on the actual situation of the interview (Erciyes, 2019). Semi-structured interviews enable researchers to obtain more detailed as well as diversity of data in the analysis process, which further enriches the research content. The purpose of the interviews was to understand in greater detail the factors that promote the learning outcomes of international students.

The interviews were mainly based on the following three questions: (1) What is your motivation and experience for studying abroad? (2) What learning outcomes did you achieve during university? (3) What factors motivated you to achieve the above learning outcomes? The participants of the semi-structured interview were 20 international students, who had experience in studying abroad and also participated in questionnaire survey; they voluntarily participated in the face-to-face interviews. Each interview lasted about 30 min and the content was sorted and classified after the interview.

The participants used some motivated terms, such as: “*I like China*,” “*I learn a lot of knowledge and experience*,” “*I gain friends and adopt the culture*,” “*It has always been my deram and I was very excited to be exposed to more advanced knowledge and gain international experience, gain professional knowledge, and learn a new language*.” The qualitative data in the semi-structured interviews was converted to quantitative data using the aggregated value number of different motivated terms used by the partacipitants in the answers to the questions above during the interviews.

### Measurement of Variables

The measurement of variables is one of the most important parts for conducting any kind of empirical research study. In this section we describe the measurement of the main items in the questionnaire. A 5-point Likert scale rating from 1 (strongly disagree) to 5 (strongly agree) was used to evaluate each item in the questionnaire. The questionnaire was composed of three sets of items (see the Appendix): learning motivation (20 items), learning experience (11 items), and learning outcomes (9 items).

#### Learning Motivation

Sample questions of this set were “*I want to develop mind and intellectual abilities*,” “*I enjoy learning and studying*,” and “*The chance to meet new people and make new friends*.” The measurement scale of learning motivation was based on the Academic Motivation Scale (AMS) developed by Vallerand et al. (1993) with a few minor modifications. The AMS is linkedto the self-determination theory and some irrelevant and inappropriate items were deleted. Additionally, the following items referred to the specific sub-hypotheses stated earlier: external regulation (7 items, Cronbach's α = 0.855), identified regulation (4 items, Cronbach's α = 0.748), achievement motivation (3 items, Cronbach's α = 0.568), knowledge motivation (3 items, Cronbach's α = 0.539), and experience stimulation (3 items, Cronbach's α = 0.531).

#### Learning Experience

Sample questions of this set were “*I have adjusted well to my social life at university*,” “*The lecturers on my course were approachable*,” “*I have been good at working independently*,” and “*It was easy to understand the rationale for course contents*.” The measurement of learning experience was based on the Course Experience Questionnaire (CEQ) (Ramsden, 1991). In this study, we adopted the Good Teaching Scale and Generic Skills Scale of CEQ to measure the course learning experience. In addition, considering the linguistic and cultural adaptation of international students, items related to adaptability have been added. Out of the initial 11 items reserved to measure learning experience, the scales showed good internal consistency reliability after removing Q7, Q10, and Q13 (Cronbach's α = 0.614, 7 items for generic skills; Cronbach's α = 0.735, 2 items for good teaching; Cronbach's α = 0.640, 2 items for adaptability).

#### Learning Outcomes

Sample questions of this set were “*I found my course very interesting*,” “*l feel that I really belong at this university*,” and “*I get satisfaction from meeting intellectual challenges and pushing my limits*.” The measurement of learning outcomes was based on Kraiger et al.'s (1993) and Jordan's (2011) research, and the College Outcome Survey Questionnaire. The two dimensions of learning outcomes (namely cognitive and non-cognitive) were also differentiated. After removing questions 2, 3, 4, 5, and 12 to reach a good internal consistency reliability, 5 items were used to for non-cognitive learning outcomes (Cronbach's α = 0.792) and 4 items for cognitive learning outcomes (Cronbach's α = 0.714).

### Data Analysis

In addition to the 40 questions (items) described above, semi-structured interviews were conducted to enrich the data and improve the validity and completeness of the empirical study. Further, combination of qualitative and quantitative methods was used to analyze the questionnaire data.

Regarding the quantitative part of the survey, some questions (items) were removed or revised to improve the reliability before data analysis. Factor analysis was conducted to explore the most relevant factors influencing learning motivation, learning experience and learning outcomes dimensions. The data was analyzed using stepwise regression with Ucinet6 software to test the main relationships. Stepwise regression was chosen to test the interaction between variables as well as the moderating effect.

In addition to the quantitative items, semi-structured interviews were conducted concerning students' motivation, learning experience, and the factors that influenced achievement of learning outcomes. The interview questions were intended to supplement and expand the quantitative data and facilitate a deeper understanding of the results. The data obtained from the semi-structured interviews were analyzed independently from the previous quantitative items, thus ensuring the reliability of the research study (Ballinger et al., 2004).

### Reliability and Validity Test

As shown in Table 2, the Cronbach's alpha value of each variable in the questionnaire ranges from 0.706 to 0.871. This means the internal consistency and scale reliability, was good. Additionally, as the questionnaires used different existing maturity scales with some modifications due to the inherent characteristics of this study, the scales content validity also had to be tested. In this regard, the Kaiser-Meyer-Olkin (KMO) Test was implemented on the learning motivation, learning experience, and learning outcomes scales. The values were 0.806, 0.694, and 0.808, respectively, indicating adequate structural validity. Table 2 shows the factors as well as reliability and validity test data.

**Table 2:** The factors and reliability/validity tests.

**Variable**	**Dimension**	**Numbers**	**Cronbach's α**	**KMO value**	**Bartlett value**	**Cumulative proportion in ANOVA %**
Learning motivation	External regulation	7	0.871	0.806	776.494	59.956
	Identified regulation	4				
	Achievement motivation	3				
	Knowledge motivation	3				
	Experience stimulation	3				
Learning experience	Generic skills	7	0.706	0.694	225.725	53.669
	Adaptability	2				
	Good teaching	2				
Learning outcomes	Non-cognitive learning outcomes	5	0.796	0.808	273.409	56.566
	Cognitive learning outcomes	4				

## Results

### Basic Statistics

Table 3 shows the basic statistical results of the study, whichaims to detail the impact of learning motivation and learning experience on learning outcomes. These results contain the calculated means, standard deviations, and correlation coefficients of all variables involved in the analysis. Table 3 provides edescriptive statistics and correlation coefficients of all the factors included in the questionnaire deployed in the study.

**Table 3:** Descriptive statistics and correlation coefficients of the factors.

	**1**	**2**	**3**	**4**	**5**	**6**	**7**	**8**	**9**	**10**
1. External regulation	1									
2. Identified regulation	0.376^**^	1								
3. Achievement motivation	0.395^**^	0.398^**^	1							
4. Knowledge motivation	0.505^**^	0.476^**^	0.402^**^	1						
5. Experience stimulation	0.366^**^	0.235^*^	0.264^**^	0.309^**^	1					
6. Generic skills	0.502^**^	0.364^**^	0.175	0.455^**^	0.047	1				
7. Adaptability	0.368^**^	0.220^*^	0.343^**^	0.445^**^	0.060	0.224^*^	1			
8. Good teaching	0.248^*^	0.157	0.181	0.051	0.239^*^	0.139	0.204^*^	1		
9. Non-cognitive learning outcomes	0.396^**^	0.232^*^	230^*^	0.433^**^	−0.019	0.516^**^	0.468^**^	0.079	1	
10. Cognitive learning outcomes	0.777^**^	0.417^**^	0.612^**^	0.574^**^	0.308^**^	0.462^**^	0.423^**^	0.259^**^	0.373^**^	1
Mean	1.789	2.626	2.159	1.937	1.852	2.388	2.091	2.348	2.291	1.891
Standard deviation	0.616	0.808	0.741	0.680	0.612	0.622	0.820	0.731	0.797	0.666

^*^p < 0.05, ^**^p < 0.01.

Is the results show that experience stimulation is weakly correlated with non-cognitive outcomes. However, all other dimensions (except experience stimulation motivation) of learning motivation have a significant positive effect on cognitive and non-cognitive outcomes. The correlation coefficient between external regulation and cognitive learning outcomes is the largest (0.777, *p* < 0.01). Experience stimulation, on the other hand, is negatively correlated with non-cognitive outcomes. Therefore, with a few exceptions, the correlation analysis shown in Table 3 provides initial evidence supporting most of the research hypotheses.

### Stepwise Regression Model

The study adopted cognitive and non-cognitive learning outcome as dependent variables, and learning motivation as an independent variable along with control variables, including gender, age, and language of the participants. Models 1 to 4 have non-cognitive learning outcomes as the dependent variable. Models 5 to 8 use cognitive outcomes as the dependent variable. Then, an adjustment variable (i.e., learning experience) was added to the model. Finally, the interaction terms of learning motivation and learning experience were inserted to verify the moderating effect of learning experience. The results are shown in Table 4.

**Table 4:** Regression analysis results.

**Variables**		**Non-cognitive learning outcomes**				**Cognitive learning outcomes**			
		**Model 1**	**Model 2**	**Model 3**	**Model 4**	**Model 5**	**Model 6**	**Model 7**	**Model 8**
Control variable	Gender	0.164	0.089	0.090	0.118	−0.111	−0.307	−0.279	−0.229
	Ages	−0.260	−0.229	−0.182	−0.202	0.079	0.204	0.290^*^	0.369^**^
	Language	0.332^***^	−0.029	0.056^**^	0.033	0.112	−0.064	−0.162	−0.079
Independent variable	External regulation (A)		0.258^**^	0.062	0.103		0.273^**^	0.167^*^	0.051
	Identified regulation (B)		0.179^*^	0.091	0.064		0.060	0.0117	0.037
	Achievement motivation (C)		0.156^*^	0.115	0.191^*^		−0.190	−0.267	−0.284
	Knowledge motivation (D)		0.275^**^	0.079	0.020		0.264^***^	0.160^*^	0.151
	Experience stimulation (E)		−0.123	−0.077	−0.089		0.028	−0.005	−0.008
Moderator variable	Generic skills (F)			0.334^**^	0.312^*^			0.108	0.211
	Adaptability (G)			0.298^**^	0.290^*^			0.206^**^	0.262^*^
	Good teaching (H)			−0.190	−0.176			0.229^***^	0.154
Interaction terms	A*F				−0.167				0.254^**^
	A*G				0.173^*^				−0.223
	A*H				−0.103				0.140
	B*F				0.001				−0.223
	B*G				−0.015				0.080
	B*H				−0.041				−0.051
	C*F				0.051				0.078
	C*G				0.088				−0.035
	C*H				0.010				−0.096
	D*F				0.134				−0.015
	D*G				−0.081				0.201^*^
	D*H				−0.124				−0.010
	E*F				−0.014				0.186^*^
	E*G				0.018				0.047
	E*H				−0.026				0.064
Maximum VIF value		1.003	1.073	1.193	1.407	1.000	1.027	1.057	1.293
*R^2^*-Value		0.051	0.260	0.402	0.538	0.005	0.162	0.233	0.476
Adjusted *R^2^*		0.014	0.190	0.325	0.379	−0.034	0.083	0.134	0.295
*ΔR^2^*		0.051	0.209	0.142	0.136	0.005	0.157	0.071	0.243
*F*-Value		1.807^*^	4.165^**^	5.627^**^	3.453^*^	0.183	2.292^*^	2.535^*^	2.692^*^

^*^p < 0.1, ^**^p < 0.05, ^***^p < 0.01.

In the models with the interaction terms inserted, the moderating effect is significant as the *R*^2^-value increases significantly (i.e., the partial regression coefficient of the interaction becomes significant). Particularly, models 1 and 5 explore the effects of the control variables on the dependent variables. In model 1, the *F*-value is 1.807 with a significance level of 0.1, *R*^2^ = 0.051, meaning that the explanatory ability is only 5.1%. The regression coefficient of language to non-cognitive learning outcomes is 0.332 and significant (*p* < 0.01). In model 5, the explanatory ability of the control variables to cognitive outcomes is clearly insufficient (*R*^2^ = 0.005), the normalized regression coefficient is not significant and the model does not pass the *F*-test. Therefore, the control variables do not affect the cognitive outcomes.

Models 2 and 6 add the independent variable to the previous model to test the impact of learning motivation on cognitive and non-cognitive learning outcomes. As shown in Table 4, model 2 has an F value of 4.165, and the overall model is significant, the *R*^2^-value is 0.260 and the model fits well. Compared with model 1, the whole interpretation ability of model 2 is improved by 20.9% (Δ*R*^2^ = 0.209). At the same time, the standardized coefficient show that the regression coefficients of external regulation and knowledge motivation are 0.258 and 0.275 (*p* < 0.05), respectively. The regression coefficients of identified regulation and achievement motivation are 0.179 and 0.156, respectively, and the *p*-values are both <0.1, thereby indicating that all four types of learning motivations had a significant positive effect on non-cognitive outcomes. Therefore, *H1* is supported.

Model 6 is significant (*F* = 2.292, *p* < 0.1). The explanatory ability is 16.2% and the coefficient of external regulation motivation is 0.273 (*p* < 0.05), while the knowledge motivation's coefficient is 0.264 (*p* < 0.01), which shows that external regulation and knowledge motivation are significantly positively correlated with cognitive learning outcomes. Therefore, *H2* is supported.

In models 3 and 7, learning motivation and learning experience are added to existing models. The F value is significant at the 0.05 level and *R*^2^-value is 0.402. The explanatory capacity of model increases by 14.2%. The results showed that the model 7 is significant at the 0.1 level, with *F* = 2.535, and the fitness is much better now (Δ*R*^2^ = 0.071, *R*^2^ = 0.233). The coefficient of good teaching in the model is 0.229 with a significance level of 0.01, indicating that good teaching is positively related to cognitive outcomes.

Models 4 and 8 include the interaction terms, and the explanatory ability on the full-effect models improves significantly (*R*^2^ = 0.538 > 0.402, *R*^2^ = 0.476 > 0.233), with significant F statistics at the 0.1 level showing that learning experience plays an effective role on the relationship between the learning motivation of international students and learning experience. Therefore, *H3* and *H4* are both supported.

### Findings of the Semi-structured Interviews

A total of 20 students participated in the semi-structured interviews, which consists of 19% of the participants in the survey. According to a summary of the interview records of the first question, we can see that most participants (17 out of 20) agree that their motivation for studying abroad is due to the desire for knowledge, job hunting, expectations of other people and international exploration. Motivation for studying abroad is the main factor determining achievement of the eventual learning outcomes. For example as can be observed from the following quotations from participants:

“*I like Chinese culture, so I came to Xi'an for study. I learned a lot of knowledge and gained a lot of friends and happiness here. It has always been my dream and motivation to study in a Chinese university. I was very excited when the dream came true”* Participant # 1.

“*The motivation for me to study in China comes from the fact that I want to be exposed to more advanced knowledge about my profession. I like the economics, so I will study hard and overachieve in the relevant professional fields of my interest”* Participant # 2.

Further, 9 out of 20 participants agreed that job hunting and employment prospects is one of the main reasons for studying in China. Their views were as follows:

“*The main reason why I choose to study in China is to master important professional knowledge and improve my language skills. I will have more opportunities to engage in my favorite career after graduation. I am now full of motivation”* Participant # 3.

“*I am majoring in civil engineering. I hope to master the knowledge of architectural structures through the study of professional courses so that I can enter a building company in the future. Now, I am actively participating in various internships and building a sound professional foundation. I hope that the school can provide me with more relevant courses”* Participant # 4.

Learning motivation comes not only from internal factors, but also from external factors. Indeed, some (6 out of 20) students use their own experiences to explain the impact of others expectations on their own learning.

“*I have been performing well since I was a child. My parents have high expectations form me, so I am strict with myself every day and eager to get their praise”* Participant # 5.

In regrad to the influence of learning experience on learning motivation and learning outcomes, many participants agreed that adaptation to the environment affects achievement of the learning outcomes. For example, an interviewee from Russia claimed that the prior experience made the student more autonomous and motivated through adapting to the new environment and knowledge. The participant said the following:

“*Before studying abroad, I had a short experience of studying in mainland China as an exchange student for three months, which helped me to adapt to the environment of China in a short period of time, and my strong communication ability help me gain many friends. I can effectively integrate knowledge and improve my learning efficiency”* Participant # 6.

Additionally, another international student stated that adapting to school life made the student more dynamic with a sense of belonging. The participant said the following:

“*When I first came to China, I was very unfamiliar with the school environment. I communicated with Chinese students about campus culture and school regulations. I quickly adapted to the school life and preferred to challenge myself and study hard. I feel that I am fully integrated into the life of the school and I have a sense of belonging”* Participant # 7.

## Discussion

**Figure 2:**
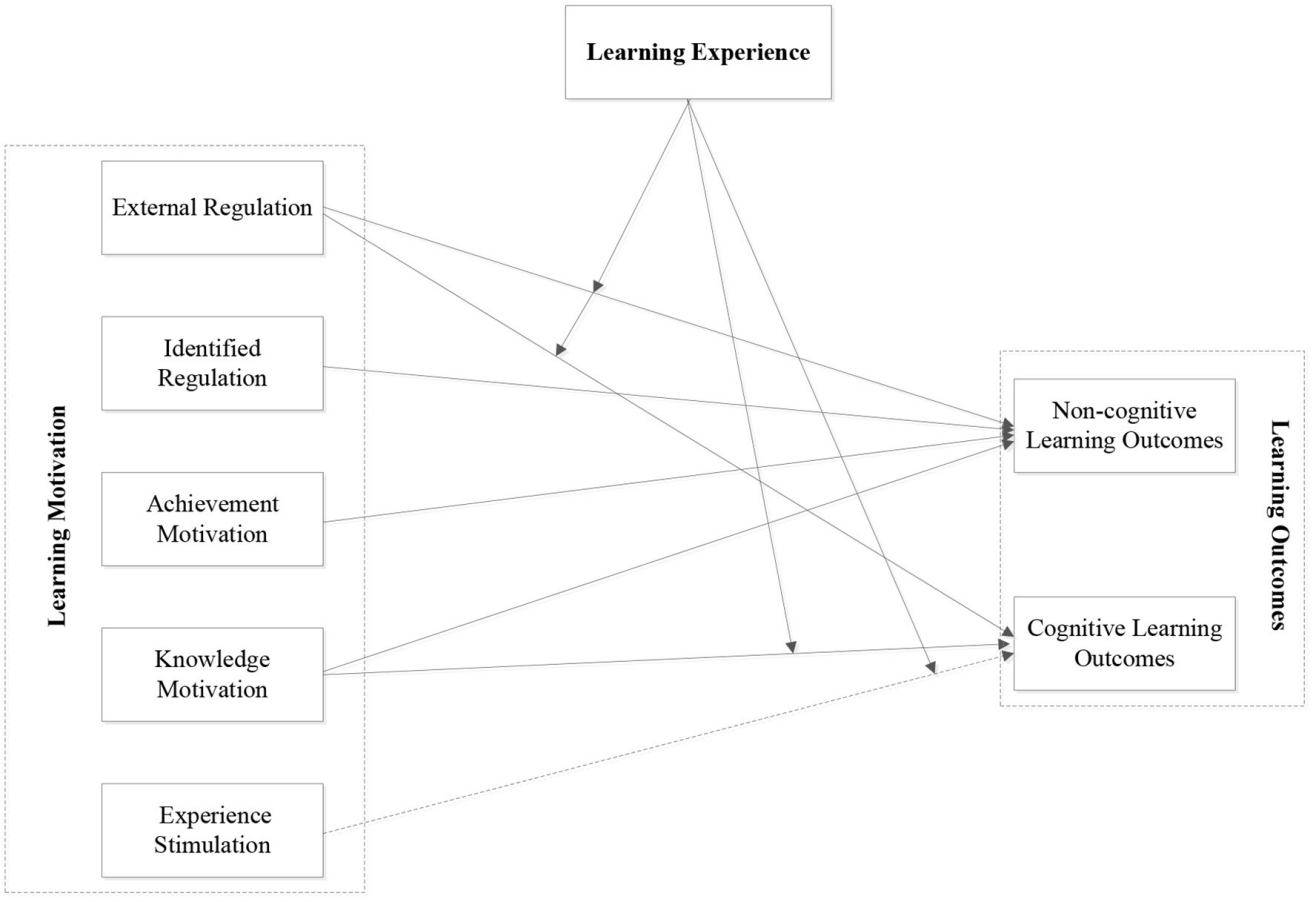
Final MEO model. The solid lines indicates significant relationships between variables, while the broken line represents that the relationship is not significant.

Figure 2 provides the final MEO model based on the above quantitative and qualitative results. The MEO model and results support the research hypotheses (namely H1, H2, H3, and H4). External regulation and knowledge motivation have the most positive effect on achievement of learning outcomes. In addition, general skills play the most positive role in the relationship between external motivation and cognitive learning outcomes compared with experience of adaptability.

Consistent with the findings of Hsieh (2014), the results show that gender does not significantly affect learning outcomes. Language ability, on the other hand, does have a positive influence on the non-cognitive outcomes. This is probably because of a lack of educators with professional English teaching abilities. But also because it is difficult for international students to completely integrate into the course study during classes, thus decreasing learning efficiency. At the same time, the narrow interpersonal communication skills involved is not conducive to the rapid adaptation to the local culture for international students, thereby greatly affecting their learning status and ultimately leading to poor study results.

In this study, external regulation and knowledge motivation is significantly related to learning outcomes. Although our results are slightly different to previous studies, the basic trends are consistent with previous research. For example, according to Fenollar et al. (2011), the impact of achievement motivation on cognitive outcomes is not significant. In contrast, highly motivated students are better at achieving good learning outcomes. Furthermore, the correlation coefficient for external regulation and learning outcomes is rather higher. This is consistent with Wentzel (2010) and is likely to be due to foreign students studying abroad mainly in pursuit of their future plans for improved career development prospects. Such students show a tendency of extrinsic motivation when they study abroad (Li and Bray, 2007; Chue and Nie, 2016). With enhanced external regulation, the persistence of students along with seriousness and cognitive engagement with learning is stronger. By comparing the learning outcomes of international students with their desired future direction, adjustments, and revisions are made to help achieve the positive development of their learning outcomes.

With regard to the relationship between knowledge motivation and learning outcomes, the results shows the positive effect of intrinsic motivation (knowledge motivation) on international students' learning outcomes. Previous studies have confirmed that compared with other types of learning motivation, intrinsic motivation is more related to students' advanced studies and good performance (Orsini et al., 2016). This is because international students have a stronger sense of self and cognitive development compared with local students. There exists a higher level of intrinsic motivation, along with more curiosity and desire for knowledge in academic activities (Olle Th et al., 2011). Under this kind of motivation, international students achieve more satisfactory learning outcomes.

The study also verified that learning experience positively regulates the correlation between learning motivation and learning outcomes. Here, as a moderator variable, learning experience increased the model's explanatory ability by 14.2%, thereby fully demonstrating its role as an effective moderator variable. The results are consistent with previous findings that learning experience plays an important role in learning activities (Haverila, 2012). Generic skills have a significantly positive moderating effect on the relationship between external regulation and cognitive outcomes. Compared with weaker generic skills, international students with rich generic skills are likely to adapt to the learning environment to improve their motivation and acquisition of knowledge and skills. Students who do not have the optimal generic skills, such as time management and planning, are likely to have no motivation and goals, may not sustain their study and thus usually drop out of university education (Kumpas, 2012). This result suggests that academic institutions could offer practical and generic skills courses to help improve the skills of international students, which will further help improve learning outcomes.

Meanwhile, the results confirm that adaptability positively regulates the relationship between external regulation and non-cognitive outcomes. Many researchers have emphasized that an active learning environment can meet the motivational needs of students in the learning process, so as to improve the overall motivation and learning outcomes (Abeysekera and Dawson, 2015; Sergis et al., 2018). Therefore, the experience of international students in adapting to the local environment (i.e., abroad) is very important. In this regard, strong and adaptive faculty members can help international students to change their learning attitude, promote the internal improvement of motivation in the learning process, thus contribute to improved attitudinal and emotional learning outcomes.

## Conclusions

This empirical study has developed a conceptual model based on the extant literature that explores the relationship between learning motivation of international students and learning outcomes with the moderating role of learning experience. The results are based on a theoretical analysis and formulated through hypothesis testing. The results show that the motivation to learn has a positive impact on learning outcomes, particularly external regulation, knowledge, identified regulation, and achievement motivation, which significantly affect non-cognitive outcomes; whereas external regulation and knowledge motivation are significantly positively correlated with cognitive outcomes. Learning experience has a moderating effect, with adaptability positively affecting the relationship between external regulation and non-cognitive outcomes—generic skills play a positive role in regulating the relationship between external regulation and cognitive outcomes.

The study contributes to the literature across a diverse field of research including knowledge management, personality-based studies, psychology, and engineering. Firstly, the study enriches and expands theoretical research and provides new ideas and perspectives for related research studies. This is because previous work mainly focused on the relationship between learning motivation and learning outcomes exclusively. The present study does not only pay attention to the effect of different learning motivations, but also refers to the synergy with learning experience, therebyovercoming some of the deficiencies of current educational research.

Secondly, the study constructs a model of learning motivation, learning experience, and learning outcomes, and explores the relationship between the dimensions of each variable with a systematic research model. The empirical research also used a combination of quantitative and qualitative analyses (i.e., mixed method research), including additional comments solicited from participants in the study for added richness. Thirdly, as there have been relatively few studies of international students studying in mainland China, this study contributes to the empirical research in this geographical area.

The research conclusions shed light on how to promote education by international students in mainland China. In the context of higher education internationalization, the number of international students coming to mainland China is continually increasing. An ability to know how to effectively stimulate learning motivation among such students is key to improve the overall quality of their academic as well as wider social performance. Indeed, motivation to learn is positively related to satisfactory learning outcomes, and consequently the character and extent of motivation should directly inform the teaching methods and processes used in class. The learning experience can effectively regulate the relationship between learning motivation and outcomes. Academic institutions need to formulate suitable incentive policies to help international students strengthen their motivation to learn as well as improve learning efficiency and achievement of learning outcomes. Furthermore, students can help themselves by improving their language skills and intercultural competences to build and augment their own learning style. The accumulation of learning experiences will also promote experiential learning as part of the overall learning process. For example, higher education institutions can increase the number of international student awards and funded grant programs to encourage more extrinsic motivation by international students and improve the learning effect. Research institutions and academicians can also offer employment and general skills courses to help foreign students increase their sense of achievement and enhance their intrinsic and extrinsic learning motivation, which can also improve the quality of international student education management.

This research study is limited by the sample studied comprising only international students in a single city of Xi'an in Shaanxi Province in China. In future research, a wider range of participants is needed to enhance the application and external validity of the conclusions. It is also noted that the study only considers the effects of learning experience on the relationship between learning motivation and learning outcomes. Future research is therefore needed to explore the mechanism of learning motivation and learning outcomes from other theoretical perspectives.

## Data Availability Statement

The original contributions presented in the study are included in the article/Supplementary Material, further inquiries can be directed to the corresponding author/s.

## Ethics Statement

Ethical review and approval was not required for the study on human participants in accordance with the local legislation and institutional requirements. Written informed consent from the patients/ participants or patients/participants legal guardian/next of kin was not required to participate in this study in accordance with the national legislation and the institutional requirements.

## Author Contributions

JZ developed the theory and wrote the article. GS helped in data analysis and results. LX reviewed the article. IK involved in copyediting and data collection. WL helped in final review process. SP helped in revision of the word and polishing the language. All authors contributed to the article and approved the submitted version.

## Funding

This research was supported by the National Social Science Fund projects of China (Grant No. 20BJY010); National Social Science Fund Post-financing projects of China (Grant No. 19FJYB017); China Sichuan-Tibet Railway Major Fundamental Science Problems Special Fund (Grant No. 71942006); China Qinghai Natural Science Foundation (Grant No. 2020-JY-736); List of Key Science and Technology Projects in China's Transportation Industry in 2018-International Science and Technology Cooperation Project (Grant Nos. 2018-GH-006 and 2019-MS5-100); Emerging Engineering Education Research and Practice Project of Ministry of Education of China (Grant No. E-GKRWJC20202914); Higher Education Teaching Reform Project in Shaanxi Province, China (Grant No. 19BZ016); Humanities and Social Sciences Research Project of the Ministry of Education of China (21XJA752003); Project of the Academy of Social Sciences of Shaanxi Province, China (2022HZ0596); Going Global Partnership: UK-China-ASEAN, Education Partnership Initiative funded by British Council (Integrated Built Environment Teaching & Learning in the Joint Curriculum Development amid Digital-Driven Industry 4.0 among China, Vietnam, and UK); International Education Research Program of Chang'an University, China, 2022 (Grant No. 300108221113); and National Natural Science Foundation of China (Grant No. 72074191).

## Conflict of Interest

The authors declare that the research was conducted in the absence of any commercial or financial relationships that could be construed as a potential conflict of interest.

## Publisher's Note

All claims expressed in this article are solely those of the authors and do not necessarily represent those of their affiliated organizations, or those of the publisher, the editors and the reviewers. Any product that may be evaluated in this article, or claim that may be made by its manufacturer, is not guaranteed or endorsed by the publisher.
