# Corrigendum: The moderating effect of learning experience on learning motivation and learning outcomes of international students

**DOI:** 10.3389/fpsyg.2022.1093960

**Published:** 2023-01-10

**Authors:** Jingxiao Zhang, Gangzhu Sun, Lin Xu, Inayat Khan, Weidong Lv, Simon P. Philbin

**Affiliations:** ^1^School of Economics and Management, Chang'an University, Xi'an, China; ^2^School of Civil Engineering, Zhengzhou University, Zhengzhou, China; ^3^School of Foreign Languages, Northwest University, Xi'an, China; ^4^School of Management, Northwest Polytechnical University, Xi'an, China; ^5^School of International Education, Chang'an University, Xi'an, China; ^6^School of Engineering, London South Bank University, London, United Kingdom

**Keywords:** international students, learning motivation, learning experience, learning outcomes, experimental study and application

In the published article, there was an error in the Correspondence section. Author Gangzhu Sun was erroneously excluded as a corresponding author. The corrected Correspondence section appears below.

“Correspondence: Jingxiao Zhang, zhangjingxiao964@126.com; Gangzhu Sun, gzsun@zzu.edu.cn; Inayat Khan, inayat@mail.nwpu.edu.cn.”

In the published article, there was also an error in the Funding section as published. Two pieces of funding were missing: International Education Research Program of Chang'an University, China, 2022 (No. 300108221113) and National Natural Science Foundation of China (No. 72074191). The correct Funding statement appears below:

