# Supplementary material for: The Moderating Effect of Learning Experience on Learning Motivation and Learning Outcomes of International Students
Source: Front Psychol. 2022 Jun 28;13:913982. doi: 10.3389/fpsyg.2022.913982 (PMC9306406; doi:10.3389/fpsyg.2022.913982)
Supplement: Supplementary file 1 [file Data_Sheet_1.docx]

**Appendices**

## Appendix I Learning Motivation Questionnaires

### Table 1 Learning Motivation Questionnaire

1.外部法规2.确定的法规。它是指个体对一个行为目标或规则进行有意识的评价，如果发现这个行为是重要的，就接纳为自我的一部分的一种动机类型。个体更多地体验到自己是行为的主人，感觉到更少的冲突。它含有更多的自主或自我决定的成分，是最具有自主性的外部动机形式。如：渴望成为一个可以说外语的人。3，成就动机4，知识动机5，体验刺激

| NO. | Item |  | Strongly agree=5 points | Agree  =4 points | Neither agree nor disagree=3 points | Disagree  =2 points | Strongly disagree=1 point |
| --- | --- | --- | --- | --- | --- | --- | --- |
| M-1 | I am interested in pursing postgraduate studies | 5 |  |  |  |  |  |
| M-2 | Want to develop a better understanding of myself | 3 |  |  |  |  |  |
| M-3 | I can success at university | 3 |  |  |  |  |  |
| M-4 | Completing this degree will increase my earning power | 1 |  |  |  |  |  |
| M-5 | Done well in school, going to university is natural to do | 2 |  |  |  |  |  |
| M-6 | All my friends were going to university | 1 |  |  |  |  |  |
| M-7 | Believe university degree will open up new opportunities for future | 5 |  |  |  |  |  |
| M-8 | Drifted into higher education | 5 |  |  |  |  |  |
| M-9 | University can give opportunity to improve self-confidence | 5 |  |  |  |  |  |
| M-10 | I want the chance to broaden horizons and face new challenges | 1 |  |  |  |  |  |
| M-11 | Degree will enable to meet education requirements for my career | 1 |  |  |  |  |  |
| M-12 | Enable me to get a good job | 1 |  |  |  |  |  |
| M-13 | I want to develop mind and intellectual abilities | 3 |  |  |  |  |  |
| M-14 | Progressing to university is what others expected | 1 |  |  |  |  |  |
| M-15 | I enjoy learning and studying | 5 |  |  |  |  |  |
| M-16 | I am attracted by the opportunities for an active social life | 5 |  |  |  |  |  |
| M-17 | This degree will help me develop knowledge and skills in my life after university | 4 |  |  |  |  |  |
| M-18 | Really want to get a university degree | 3 |  |  |  |  |  |
| M-19 | Coming to university affords me three more years to decide what I really want to do |  |  |  |  |  |  |
| M-20 | I want to be a better educated person | 3 |  |  |  |  |  |
| M-21 | The chance to meet new people and make new friends | 1 |  |  |  |  |  |

*Note.* If the participants agree with the opinion, then tick below. Variable is measured by students’ responses to the item, rated on a 5-point Likert scale from 1, strongly disagree, to 5, strongly agree (Strongly agree=5 points;Agree=4 points; Neither agree nor disagree=3 points; Disagree=2 points; Strongly disagree=1 point). The measurements in Table 2, Table 3 are the same as in Table 1.M=Motivation, EPR= Experience, LO=Leaning Outcomes.

Strongly disagree

Disagree

Neither agree nor disagree

Agree

Strongly agree

## Appendix II Learning Experience Questionnaires

### Table 2 Learning Experience Questionnaire

Generic Skills Adaptability Good Teaching

| NO. | Item |  | Strongly agree=5 points | Agree  =4 points | Neither agree nor disagree=3 points | Disagree  =2 points | Strongly disagree=1 point |
| --- | --- | --- | --- | --- | --- | --- | --- |
| EPR-1 | I find it easy to manage time and workload | 1 |  |  |  |  |  |
| EPR-2 | I worked consistently throughout my time | 1 |  |  |  |  |  |
| EPR-3 | I have been good at working independently | 1 |  |  |  |  |  |
| EPR-4 | Expected to become independent learners too quickly |  |  |  |  |  |  |
| EPR-5 | University timetables should be more convenient for students | 2 |  |  |  |  |  |
| EPR-6 | Sometimes felt pressurized by financial worries |  |  |  |  |  |  |
| EPR-7 | I have adjusted well to academic demands of university | 2 |  |  |  |  |  |
| EPR-8 | I have adjusted well to my social life at university | 2 |  |  |  |  |  |
| EPR-9 | It was easy to understand the rational for course contents | 2 |  |  |  |  |  |
| EPR-10 | The workload on my course was heavy | 2 |  |  |  |  |  |
| EPR-11 | The academic demands of work on my course were difficult | 2 |  |  |  |  |  |
| EPR-12 | The physical environment of university is pleasant | 2 |  |  |  |  |  |
| EPR-13 | The lecturers on my course were good at explaining things | 3 |  |  |  |  |  |
| EPR-14 | The lecturers on my course were approachable | 3 |  |  |  |  |  |

## Appendix III Leaning Outcomes Questionnaires

### Table 3 Leaning Outcomes Questionnaire

| NO. | Item | Strongly agree=5 points | Agree  =4 points | Neither agree nor disagree=3 points | Disagree  =2 points | Strongly disagree=1 points |
| --- | --- | --- | --- | --- | --- | --- |
| LO-1 | I found my course very interesting |  |  |  |  |  |
| LO-2 | I only want to study topics which relevant to my career |  |  |  |  |  |
| LO-3 | Inherent abilities is the biggest factor in academic success |  |  |  |  |  |
| LO-4 | I did only the minimum of work which was required of me |  |  |  |  |  |
| LO-5 | The lecturers stimulated my interest in my career |  |  |  |  |  |
| LO-6 | I have enjoyed my studies so far at this university |  |  |  |  |  |
| LO-7 | I found it difficult to get motivated to work on course |  |  |  |  |  |
| LO-8 | I want to get high marks at university |  |  |  |  |  |
| LO-9 | I know how well I’m doing in order to feel motivated to work |  |  |  |  |  |
| LO-10 | I’m keen to learn about new aspects of my subject and to explore new ideas |  |  |  |  |  |
| LO-11 | I find it easy to talk about university with friends and families |  |  |  |  |  |
| LO-12 | I get satisfaction from meeting intellectual challenges and pushing my limits |  |  |  |  |  |
| LO-13 | Course boring but will stick with it because l want a good job |  |  |  |  |  |
| LO-14 | l feel that l really belong at this university |  |  |  |  |  |

## Appendix IV Demographic Information

### Table 4 Demographic Information

| NO. | Level of study | Language | Nationality | Age | Gender | Course | Transfer course | Last qualification | Sponsor |
| --- | --- | --- | --- | --- | --- | --- | --- | --- | --- |
| 1 | 2 | 2 | ZAMBIAN | 2 | 1 | Civil engineering | 2 | 1 | 2 |
| 2 | 2 | 2 | ZAMBIAN | 2 | 1 | Civil engineering | 2 | 2 | 2 |
| 3 | 2 | 2 | ZAMBIAN | 2 | 1 | Civil engineering | 2 | 2 | 2 |
| 4 | 2 | 2 | ZAMBIAN | 3 | 1 | Civil engineering | 2 | 2 | 3 |
| 5 | 2 | 2 | ZAMBIAN | 3 | 2 | Civil engineering | 2 | 1 | 2 |
| 6 | 2 | 2 | ZAMBIAN | 3 | 1 | Civil engineering | 2 | 1 | 2 |
| 7 | 2 | 1、2 | PASKISTANI | 3 | 1 | Civil | 2 | 2 | 2 |
| 8 | 2 | 1 | UONGDIA | 3 | 2 | Communication engineer | 2 | 4 | 4 |
| 9 | 2 | 1 | NEPALESE | 3 | 2 | Hourism management | 2 | 4 | 4 |
| 10 | 3 | 1 | SOUTH AFRICAN | 3 | 2 | Mba major | 2 | 4 | 5 CSC |
| 11 | 1 | 1 | MONGOLIA | 3 | 2 | Environmental engineer | 2 | 4 | 4 |
| 12 | 3 | 1 | MONGOLIA | 3 | 1 | electrical engineering | 2 | 4 | 4 |
| 13 | 3 | 2 | MONGOLIA | 3 | 2 | electrical engineering | 2 | 4 | 4 |
| 14 | 3 | 1 | NEPAL | 4 | 1 | vehicle engineering | 2 | 4 | 4 |
| 15 | 2 | 2 | TANZANIA | 1 | 1 | International trade | 2 | 1 | 2 |
| 16 | 2 | 2 | ZAMBIAN | 2 | 1 | Computer science | 2 | 2 | 2 |
| 17 | 2 | 2 | CAMEROON | 3 | 1 | Civil engineering | 1 | 2 | 2 |
| 18 | 2 | 1、2 | TANZANIA | 3 | 1 | Computer science | 1 | 3 | 2 |
| 19 | 3 | 2 | TANZANIA | 4 | 1 | IT | 2 | 1 | 2 |
| 20 | 3 | 2 | EGYPTIAN | 4 | 1 | IT | 1 | 1 | 5 CSC |
| 21 | 3 | 2 | ETHIOPIAN | 4 | 1 | GIS | 2 | 1 | 4 |
| 22 | 3 | 2 | TANZANIAN | 3 | 2 | MBA | 2 | 1 | 2 |
| 23 | 3 | 1 | NEPAL | 3 | 1 | Computer science and technology | 2 | 4 | 5 CSC |
| 24 | 2 | 2 | TANZANIAN | 4 | 1 | HRM | 2 | 4 | 2 |
| 25 | 2 | 2 | TANZANIAN | 2 | 2 | Economic and International Trade | 2 | 1 | 2 |
| 26 | 3 | 2 | TANZANIAN | 3 | 2 | MBA | 2 | 4 | 2 |
| 27 | 2 | 2 | SUDAN | 2 | 2 | Bussiness Administration | 2 | 2 | 2 |
| 28 | 3 | 1 | LAOS | 4 | 2 | Chinese language | 2 | 2 | 5 CSC |
| 29 | 2 | 2 | NEPAL | 4 | 2 | MBA | 2 | 2 | 2 |
| 30 | 3 | 1 | UZBEKISTAN | 3 | 1 | Tourism Management | 2 | 4 | 4 |
| 31 | 2 | 2 | ZIMBABWEAN | 2 | 2 | Computer science | 2 | 1 | 3 |
| 32 | 2 | 2 | TANZANIAN | 2 | 2 | International Economic and Trade | 2 | 1 | 2 |
| 33 | 2 | 2 | UGANDAN | 3 | 1 | Civil engineering | 2 | 1 | 2 |
| 34 | 2 | 2 | ZAMBIAN | 3 | 1 | Electronic information engineering | 2 | 2 | 2 |
| 35 | 2 | 2 | TANZANIA | 3 | 2 | International Economic and Trade | 2 | 1 | 2 |
| 36 | 2 | 2 | UGANDAN | 1 | 1 | Civil engineering | 2 | 1 | 2 |
| 37 | 2 | 2 | ZAMBIAN | 3 | 1 | International Computer Technology | 2 | 1 | 2 |
| 38 | 2 | ？ | DUBAI | 3 | 1 | International Computer Technology | 2 | 4 | 5 |
| 39 | 3 | 2 | TANZANIAN | 3 | 2 | English | 1 | 3 | 2 |
| 40 | 2 | 2 | ZAMBIAN | 3 | 2 | International Computer Technology | 2 | 3 | 1 |
| 41 | 2 | 2 | ZAMBIAN | 4 | 1、2 | Civil engineering | 2 | 4 | 2 |
| 42 | 2 | 1 | ZAMBIAN | 3 | 1 | Civil engineering | 2 | 1 | 2 |
| 43 | 2 | 2 | ZAMBIAN | 3 | 2 | Architecture | 1 | 1 | 2 |
| 44 | 2 | 2 | PAKISTANI | 3 | 1 | Civil engineering | 2 | 4 | 2 |
| 45 | 2 | 2 | PAKISTAN | 3 | 1 | Civil engineering | 2 | 1 | 2 |
| 46 | 2 | 2 | PAKISTANI | 3 | 1 | Civil engineering | 2 | 4 | 2 |
| 47 | 2 | 2 | SOUTH SUDANESE | 3 | 1 | Civil engineering | 2 | 2 | 2 |
| 48 | 2 | 2 | PAKISTAN | 3 | 1 | Civil engineering | 2 | 1 | 2 |
| 49 | 2 | 2 | CAMEROON | 3 | 1 | Civil engineering | 2 | 4 | 2 |
| 50 | 2 | 2 | CONGOLESE | 3 | 1 | Civil engineering | 2 | 4 | 2 |
| 51 | 2 | 2 | ZAMBIAN | 2 | 2 | Civil engineering | 2 | 2 | 2 |
| 52 | 2 | 2 | ZAMBIAN | 3 | 1 | Civil engineering | 2 | 4 | 2 |
| 53 | 2 | 2 | PAKISTAN | 3 | 1 | Civil engineering | 2 | 3 | 1 |
| 54 | 2 | 2 | PAKISTAN | 2 | 1 | Civil engineering | 1 | 4 | 2 |
| 55 | 2 | 2 | PAKISTAN | 2 | 1 | Civil engineering | 2 | 4 | 2 |
| 56 | 2 | 2 | CAMEROON | 3 | 1 | Electro-mechanic | 2 | 2 | 2 |
| 57 | 2 | 2 | ZAMBIAN | 3 | 1 | Civil engineering | 2 | 2 | 2 |
| 58 | 2 | 2 | SOUTH SUDAN | 3 | 1 | Civil engineering | 2 | 2 | 2 |
| 59 | 2 | 2 | ZAMBIAN | 3 | 1 | Electronic inpomation eng | 2 | 2 | 2 |
| 60 | 2 | 2 | PAKISTANI | 3 | 1 | Civil engineering | 2 | 4 | 2 |
| 61 | 2 | 2 | PAKISTAN | 3 | 1 | Civil engineering | 2 | 4 | 2 |
| 62 | 2 | 2 | ZIMBABWEAN | 3 | 1 | Civil engineering | 2 | 1 | 2 |
| 63 | 2 | 2 | ZIMBABWEAN | 3 | 1 | Civil university | 2 | 1 | 2 |
| 64 | 2 | 2 | ZAMBIAN | 3 | 2 | INT TRAED & ECONOMICS | 2 | 2 | 2 |
| 65 | 2 | 2 | NAMIBIAN | 4 | 1 | Computer science | 2 | 4 | 2 |
| 66 | 2 | 2 | TANZANIAN | 3 | 1 | International Trade and Economics | 2 | 4 | 1 |
| 67 | 2 | 2 | ECUADOR | 2 | 2 | INT TRAED ECONOMICS | 2 | 1 | 2 |
| 68 | 2 | 2 | ECUADOR | 3 | 2 | International Trade and Economics | 2 | 1 | 2 |
| 69 | 2 | 2 | PAKISTANI | 4 | 1 | BBA | 2 | 4 | 2 |
| 70 | 3 | 2 | TANZANIA | 2 | 1 | Computer science | 1 | 2 | 2 |
| 71 | 2 | 2 | ZIMBABWEAN | 3 | 1 | Civil engineering | 2 | 1 | 2 |
| 72 | 2 | 2 | UGANDAN | 3 | 1 | Civil engineering | 2 | 1 | ？ |
| 73 | 2 | 2 | ZAMBIAN | 2 | 2 | Civil engineering | 2 | 2 | 2 |
| 74 | 2 | 2 | ZAMBIAN | 2 | 1 | Civil engineering | 2 | 2 | 2 |
| 75 | 2 | 3 Arabic | BALISAINI | 3 | 2 | Civil engineering | 2 | 1 | 2 |
| 76 | 2 | 2 | BAHRAIN | 3 | 2 | Medicine | 2 | 1 | 2 |
| 77 | 2 | 2 | PAKISTANI | 3 | 2 | MBBS | 2 | 1 | 2 |
| 78 | 2 | 2 | PAKISTANI | 3 | 1 | Chinese language | 2 | 1 | 2 |
| 79 | 2 | 2 | BAHRAIUI | 3 | 1 | Civil engineering | 2 | 1 | 2 |
| 80 | 2 | 2 | PAKISTANI | 3 | 1 | Civil engineering | 2 | 4 | 2 |
| 81 | 2 | 2 | PAKISTAN | 3 | 1 | Civil engineering | 2 | 4 | 2 |
| 82 | 2 | 2 | CAMEROON | 3 | 1 | Civil engineering | 2 | 4 | 2 |
| 83 | 2 | 2 | ZAMBIAN | 2 | 2 | Civil engineering | 2 | 2 | 2 |
| 84 | 2 | 2 | CONGOLESE | 3 | 1 | Civil engineering | 2 | 4 | 2 |
| 85 | 2 | 2 | ZAMBIAN | 3 | 2 | Civil engineering | 2 | 4 | 2 |
| 86 | 2 | 2、3 French | RWANDESE | 3 | 1 | Civil engineering | 2 | 1 | 4 |
| 87 | 2 | 2 | PAKISTAN | 3 | 1 | Civil engineering | 2 | 4 | 2 |
| 88 | 2 | 2 | PAKISTAN | 3 | 1 | Civil engineering | 2 | 4 | 2 |
| 89 | 2 | 1 | PAKISTAN | 3 | 1 | Civil engineering | 2 | 4 | 2 |
| 90 | 2 | 2 | PAKISTAN | 3 | 1 | Civil engineering | 2 | 1 | 2 |
| 91 | 2 | 2 | SOUTH SUDANESE | 3 | 1 | Civil engineering | 2 | 2 | 2 |
| 92 | 2 | 2 | PAKISTAN | 3 | 1 | BSC Civil engineering | 2 | 1 | 2 |
| 93 | 2 | 2 | PAKISTANI | 5 | 1 | Chinese language | 2 | 4 | 2 |
| 94 | 2 | 2 | PAKISTAN | 3 | 2 | Civil engineering | 2 | 1 | 2 |
| 95 | 2 | 2 | ZAMBIAN | 2 | 1 | Civil engineering | 2 | 2 | 2 |
| 96 | 2 | 2 | CAMEROON | 4 | 1 | Civil engineering | 2 | 1 | 2 |
| 97 | 2 | 2 | CAMEROON | 4 | 1 | Civil engineering | 2 | 1 | 2 |
| 98 | 2 | 2 | SOUTH SUDANESE | 3 | 1 | Civil engineering | 2 | 2 | 2 |
| 99 | 2 | 2 | ZAMBIAN | 2 | 1 | Civil engineering | 2 | 1 | 2 |
| 100 | 2 | 2 | ZAMBIAN | 2 | 1 | Civil engineering | 2 | 1 | 2 |
| 101 | 2 | 2 | ZAMBIAN | 3 | 2 | Civil engineering | 2 | 2 | 2 |
| 102 | 2 | 2 | PAKISTAN | 2 | 2 | BSC Civil engineering | 2 | 1 | 2 |
| 103 | 2 | 2 | ZAMBIAN | 3 | 2 | Civil engineering | 2 | 2 | 2 |
| 104 | 2 | 2 | ZAMBIAN | 3 | 2 | Civil engineering | 2 | 2 | 2 |
| 105 | 2 | 2 | ZAMBIAN | 3 | 1 | Civil engineering | 2 | 2 | 3 |

## Appendix V Learning Motivation Questionnaire Data

### Table 5 Learning Motivation Questionnaire Data

| NO. | M-1 | M-2 | M-3 | M-4 | M-5 | M-6 | M-7 | M-8 | M-9 | M-10 | M-11 | M-12 | M-13 | M-14 | M-15 | M-16 | M-17 | M-18 | M-19 | M-20 | M-21 |
| --- | --- | --- | --- | --- | --- | --- | --- | --- | --- | --- | --- | --- | --- | --- | --- | --- | --- | --- | --- | --- | --- |
| 1 | 2 | 2 | 1 | 4 | 4 | 4 | 3 | 2 | 2 | 2 | 2 | 2 | 2 | 1 | 1 | 1 | 1 | 1 | 1 | 1 | 1 |
| 2 | 1 | 4 | 4 | 4 | 4 | 2 | 4 | 4 | 4 | 2 | 4 | 4 | 4 | 2 | 4 | 4 | 4 | 4 | 3 | 4 | 2 |
| 3 | 1 | 3 | 3 | 2 | 4 | 3 | 2 | 3 | 5 | 1 | 2 | 3 | 3 | 2 | 2 | 3 | 3 | 3 | 2 | 3 | 3 |
| 4 | 2 | 2 | 2 | 2 | 4 | 2 | 3 | 2 | 3 | 2 | 3 | 2 | 2 | 1 | 2 | 3 | 2 | 3 | 3 | 3 | 2 |
| 5 | 1 | 2 | 2 | 1 | 5 | 2 | 1 | 2 | 1 | 2 | 3 | 2 | 2 | 2 | 3 | 1 | 2 | 1 | 5 | 2 | 2 |
| 6 | 1 | 3 | 3 | 2 | 4 | 1 | 1 | 3 | 4 | 5 | 3 | 2 | 5 | 3 | 3 | 1 | 2 | 3 | 5 | 4 | 5 |
| 7 | 1 | 4 | 2 | 2 | 4 | 4 | 2 | 2 | 2 | 2 | 2 | 2 | 4 | 2 | 2 | 2 | 2 | 2 | 4 | 4 | 2 |
| 8 | 2 | 2 | 2 | 2 | 2 | 2 | 2 | 1 | 5 | 5 | 1 | 3 | 2 | 2 | 1 | 4 | 5 | 1 | 2 | 2 | 3 |
| 9 | 2 | 2 | 2 | 3 | 3 | 2 | 5 | 3 | 3 | 5 | 3 | 4 | 4 | 2 | 3 | 3 | 2 | 2 | 3 | 3 | 2 |
| 10 | 1 | 2 | 1 | 2 | 4 | 4 | 2 | 3 | 2 | 2 | 2 | 2 | 3 | 2 | 3 | 3 | 2 | 2 | 4 | 4 | 3 |
| 11 | 1 | 2 | 4 | 1 | 4 | 2 | 1 | 2 | 4 | 3 | 3 | 2 | 2 | 2 | 2 | 2 | 2 | 3 | 4 | 2 | 2 |
| 12 | 1 | 3 | 2 | 2 | 3 | 2 | 1 | 2 | 2 | 2 | 2 | 2 | 2 | 2 | 3 | 2 | 1 | 2 | 2 | 2 | 2 |
| 13 | 1 | 5 | 2 | 4 | 4 | 2 | 2 | 4 | 2 | 2 | 1 | 3 | 2 | 4 | 4 | 3 | 2 | 2 | 2 | 2 | 4 |
| 14 | 2 | 3 | 1 | 3 | 2 | 2 | 2 | 4 | 5 | 2 | 3 | 3 | 1 | 1 | 3 | 3 | 1 | 2 | 3 | 1 | 2 |
| 15 | 1 | 3 | 2 | 2 | 2 | 2 | 1 | 1 | 2 | 3 | 3 | 2 | 3 | 2 | 4 | 2 | 2 | 2 | 4 | 3 | 3 |
| 16 | 1 | 2 | 1 | 1 | 2 | 2 | 1 | 1 | 2 | 2 | 1 | 2 | 1 | 1 | 1 | 3 | 2 | 2 | 3 | 2 | 2 |
| 17 | 1 | 1 | 3 | 3 | 4 | 3 | 1 | 2 | 3 | 2 | 2 | 2 | 2 | 2 | 2 | 1 | 1 | 3 | 3 | 2 | 1 |
| 18 | 2 | 2 | 1 | 2 | 2 | 2 | 1 | 2 | 1 | 1 | 3 | 4 | 2 | 1 | 3 | 2 | 3 | 1 | 2 | 2 | 1 |
| 19 | 1 | 2 | 1 | 1 | 4 | 1 | 1 | 2 | 1 | 3 | 3 | 3 | 3 | 1 | 2 | 2 | 2 | 2 | 4 | 2 | 1 |
| 20 | 4 | 2 | 1 | 4 | 4 | 4 | 3 | 2 | 2 | 4 | 2 | 4 | 3 | 4 | 4 | 4 | 4 | 3 | 4 | 2 | 1 |
| 21 | 1 | 1 | 2 | 3 | 4 | 2 | 2 | 1 | 4 | 3 | 2 | 2 | 5 | 2 | 2 | 2 | 3 | 2 | 4 | 4 | 2 |
| 22 | 2 | 2 | 3 | 3 | 4 | 4 | 2 | 4 | 2 | 4 | 2 | 3 | 4 | 2 | 4 | 2 | 5 | 4 | 2 | 5 | 2 |
| 23 | 1 | 4 | 3 | 1 | 5 | 2 | 2 | 2 | 2 | 5 | 5 | 2 | 4 | 2 | 3 | 2 | 2 | 3 | 4 | 3 | 3 |
| 24 | 1 | 4 | 1 | 2 | 3 | 1 | 2 | 1 | 2 | 1 | 2 | 2 | 2 | 2 | 2 | 2 | 1 | 3 | 2 | 2 | 1 |
| 25 | 1 | 1 | 1 | 2 | 2 | 1 | 2 | 2 | 2 | 2 | 3 | 2 | 2 | 1 | 2 | 1 | 2 | 2 | 2 | 2 | 2 |
| 26 | 3 | 4 | 2 | 3 | 2 | 2 | 2 | 3 | 2 | 5 | 2 | 3 | 2 | 2 | 2 | 1 | 1 | 1 | 3 | 3 | 2 |
| 27 | 1 | 2 | 1 | 2 | 2 | 1 | 1 | 1 | 2 | 2 | 1 | 4 | 2 | 2 | 2 | 4 | 3 | 2 | 2 | 2 | 2 |
| 28 | 2 | 2 | 2 | 2 | 4 | 2 | 2 | 3 | 2 | 3 | 4 | 4 | 3 | 2 | 2 | 2 | 3 | 2 | 4 | 2 | 3 |
| 29 | 2 | 4 | 2 | 1 | 4 | 1 | 1 | 2 | 1 | 2 | 2 | 1 | 2 | 2 | 4 | 2 | 2 | 1 | 4 | 3 | 2 |
| 30 | 1 | 3 | 1 | 1 | 3 | 1 | 1 | 2 | 1 | 1 | 1 | 1 | 1 | 1 | 2 | 2 | 1 | 1 | 2 | 2 | 1 |
| 31 | 1 | 5 | 2 | 2 | 3 | 2 | 2 | 2 | 2 | 2 | 2 | 2 | 2 | 1 | 2 | 2 | 2 | 2 | 2 | 2 | 2 |
| 32 | 1 | 1 | 1 | 2 | 4 | 2 | 1 | 1 | 1 | 1 | 2 | 2 | 1 | 1 | 1 | 1 | 1 | 2 | 2 | 1 | 2 |
| 33 | 1 | 2 | 2 | 2 | 2 | 2 | 1 | 1 | 3 | 1 | 2 | 2 | 2 | 2 | 2 | 2 | 2 | 2 | 2 | 2 | 2 |
| 34 | 2 | 2 | 2 | 1 | 4 | 2 | 2 | 1 | 2 | 2 | 2 | 3 | 2 | 2 | 2 | 2 | 2 | 2 | 3 | 2 | 2 |
| 35 | 1 | 3 | 2 | 1 | 5 | 3 | 1 | 2 | 2 | 4 | 1 | 2 | 5 | 1 | 2 | 2 | 2 | 2 | 4 | 3 | 3 |
| 36 | 1 | 3 | 2 | 1 | 5 | 3 | 1 | 2 | 2 | 4 | 1 | 2 | 5 | 1 | 2 | 2 | 2 | 2 | 4 | 3 | 3 |
| 37 | 2 | 2 | 2 | 4 | 4 | 2 | 2 | 2 | 2 | 2 | 2 | 3 | 3 | 2 | 2 | 3 | 2 | 2 | 2 | 2 | 2 |
| 38 | 1 | 4 | 1 | 1 | 2 | 1 | 1 | 2 | 3 | 1 | 1 | 2 | 1 | 1 | 1 | 1 | 1 | 1 | 3 | 2 | 2 |
| 39 | 1 | 2 | 2 | 1 | 4 | 1 | 1 | 2 | 1 | 1 | 2 | 1 | 3 | 1 | 2 | 2 | 2 | 2 | 2 | 2 | 2 |
| 40 | 3 | 4 | 3 | 3 | 3 | 4 | 3 | 2 | 3 | 4 | 3 | 3 | 2 | 2 | 3 | 2 | 2 | 3 | 4 | 4 | 2 |
| 41 | 3 | 3 | 2 | 3 | 3 | 2 | 2 | 3 | 2 | 2 | 3 | 3 | 2 | 2 | 2 | 2 | 2 | 2 | 3 | 3 | 2 |
| 42 | 1 | 2 | 2 | 3 | 4 | 1 | 1 | 1 | 1 | 1 | 4 | 2 | 3 | 1 | 4 | 2 | 2 | 2 | 3 | 2 | 1 |
| 43 | 1 | 4 | 1 | 1 | 4 | 2 | 1 | 1 | 2 | 2 | 4 | 2 | 2 | 2 | 2 | 2 | 2 | 2 | 4 | 2 | 2 |
| 44 | 1 | 4 | 1 | 2 | 2 | 1 | 1 | 1 | 1 | 2 | 2 | 2 | 2 | 2 | 1 | 2 | 2 | 1 | 1 | 4 | 1 |
| 45 | 1 | 4 | 1 | 1 | 4 | 1 | 1 | 1 | 1 | 1 | 3 | 2 | 2 | 1 | 2 | 2 | 1 | 1 | 3 | 4 | 3 |
| 46 | 1 | 1 | 1 | 2 | 5 | 2 | 1 | 1 | 2 | 2 | 2 | 1 | 2 | 1 | 3 | 1 | 1 | 1 | 4 | 3 | 1 |
| 47 | 1 | 3 | 1 | 1 | 3 | 3 | 2 | 4 | 4 | 3 | 1 | 2 | 3 | 1 | 1 | 1 | 2 | 1 | 5 | 4 | 2 |
| 48 | 1 | 2 | 1 | 1 | 4 | 1 | 1 | 1 | 1 | 1 | 2 | 1 | 4 | 1 | 1 | 1 | 2 | 1 | 4 | 4 | 2 |
| 49 | 1 | 2 | 3 | 2 | 4 | 1 | 1 | 1 | 2 | 2 | 2 | 2 | 2 | 1 | 1 | 2 | 1 | 2 | 4 | 1 | 1 |
| 50 | 1 | 4 | 1 | 2 | 4 | 1 | 2 | 2 | 2 | 2 | 3 | 3 | 2 | 2 | 3 | 2 | 2 | 2 | 4 | 3 | 2 |
| 51 | 2 | 2 | 2 | 2 | 2 | 2 | 1 | 1 | 1 | 1 | 2 | 2 | 2 | 2 | 3 | 2 | 2 | 2 | 2 | 2 | 2 |
| 52 | 1 | 2 | 1 | 2 | 3 | 1 | 2 | 2 | 2 | 2 | 4 | 3 | 2 | 2 | 2 | 2 | 2 | 1 | 1 | 2 | 2 |
| 53 | 2 | 4 | 4 | 2 | 3 | 1 | 2 | 1 | 1 | 2 | 1 | 2 | 3 | 1 | 2 | 2 | 1 | 2 | 1 | 2 | 2 |
| 54 | 1 | 3 | 1 | 1 | 2 | 1 | 1 | 1 | 1 | 1 | 3 | 1 | 2 | 1 | 2 | 1 | 1 | 2 | 2 | 2 | 1 |
| 55 | 1 | 1 | 1 | 1 | 3 | 1 | 1 | 1 | 2 | 2 | 1 | 2 | 1 | 1 | 3 | 1 | 1 | 5 | 2 | 2 | 1 |
| 56 | 2 | 1 | 1 | 1 | 2 | 1 | 1 | 1 | 1 | 1 | 1 | 1 | 1 | 1 | 2 | 1 | 1 | 2 | 2 | 1 | 3 |
| 57 | 1 | 2 | 3 | 2 | 4 | 3 | 2 | 1 | 2 | 2 | 2 | 1 | 3 | 1 | 2 | 2 | 2 | 1 | 4 | 1 | 1 |
| 58 | 2 | 2 | 2 | 2 | 3 | 2 | 2 | 2 | 2 | 2 | 3 | 2 | 3 | 2 | 2 | 2 | 2 | 2 | 2 | 3 | 3 |
| 59 | 2 | 3 | 1 | 1 | 2 | 3 | 2 | 2 | 2 | 1 | 2 | 1 | 2 | 2 | 3 | 2 | 1 | 2 | 3 | 3 | 2 |
| 60 | 1 | 2 | 2 | 2 | 2 | 2 | 1 | 1 | 3 | 1 | 2 | 2 | 2 | 2 | 2 | 2 | 2 | 2 | 2 | 2 | 1 |
| 61 | 1 | 1 | 1 | 1 | 2 | 1 | 1 | 2 | 1 | 1 | 1 | 1 | 1 | 1 | 1 | 1 | 1 | 1 | 4 | 1 | 1 |
| 62 | 1 | 2 | 2 | 1 | 4 | 2 | 1 | 2 | 1 | 3 | 3 | 1 | 3 | 1 | 4 | 2 | 2 | 1 | 4 | 3 | 2 |
| 63 | 1 | 2 | 3 | 3 | 3 | 1 | 3 | 3 | 3 | 1 | 2 | 3 | 4 | 2 | 4 | 3 | 1 | 3 | 3 | 4 | 1 |
| 64 | 1 | 1 | 1 | 2 | 4 | 2 | 2 | 1 | 1 | 3 | 3 | 5 | 5 | 1 | 2 | 3 | 2 | 2 | 2 | 2 | 2 |
| 65 | 1 | 2 | 1 | 2 | 2 | 2 | 1 | 1 | 1 | 2 | 1 | 1 | 2 | 1 | 3 | 1 | 1 | 1 | 5 | 2 | 3 |
| 66 | 1 | 1 | 1 | 2 | 1 | 1 | 1 | 2 | 2 | 1 | 1 | 1 | 2 | 1 | 1 | 2 | 1 | 1 | 1 | 2 | 1 |
| 67 | 1 | 2 | 2 | 1 | 4 | 1 | 2 | 2 | 1 | 1 | 1 | 1 | 2 | 1 | 2 | 3 | 1 | 2 | 4 | 3 | 1 |
| 68 | 1 | 2 | 2 | 1 | 3 | 1 | 1 | 1 | 1 | 1 | 1 | 2 | 2 | 1 | 2 | 2 | 2 | 1 | 4 | 3 | 3 |
| 69 | 2 | 4 | 2 | 1 | 4 | 2 | 1 | 1 | 1 | 1 | 4 | 1 | 2 | 1 | 2 | 1 | 1 | 1 | 3 | 1 | 3 |
| 70 | 1 | 5 | 2 | 1 | 5 | 1 | 1 | 1 | 2 | 1 | 1 | 1 | 2 | 1 | 3 | 2 | 2 | 1 | 1 | 2 | 1 |
| 71 | 1 | 3 | 1 | 2 | 2 | 1 | 2 | 3 | 1 | 2 | 3 | 2 | 2 | 2 | 2 | 2 | 1 | 1 | 2 | 1 | 1 |
| 72 | 2 | 1 | 2 | 4 | 2 | 2 | 3 | 4 | 2 | 2 | 2 | 4 | 2 | 2 | 2 | 2 | 2 | 1 | 2 | 2 | 2 |
| 73 | 1 | 1 | 1 | 3 | 2 | 2 | 2 | 1 | 2 | 2 | 2 | 3 | 2 | 1 | 2 | 3 | 3 | 2 | 4 | 2 | 2 |
| 74 | 1 | 1 | 1 | 1 | 1 | 1 | 1 | 1 | 1 | 1 | 1 | 1 | 1 | 1 | 1 | 1 | 1 | 1 | 1 | 1 | 1 |
| 75 | 1 | 1 | 1 | 1 | 5 | 1 | 1 | 2 | 2 | 2 | 1 | 1 | 1 | 1 | 1 | 1 | 1 | 1 | 4 | 1 | 1 |
| 76 | 1 | 4 | 1 | 1 | 3 | 1 | 1 | 1 | 1 | 1 | 2 | 1 | 1 | 1 | 1 | 1 | 1 | 1 | 2 | 2 | 1 |
| 77 | 1 | 2 | 1 | 1 | 2 | 2 | 2 | 2 | 2 | 2 | 2 | 3 | 2 | 2 | 2 | 2 | 2 | 2 | 2 | 2 | 2 |
| 78 | 2 | 4 | 3 | 3 | 4 | 2 | 2 | 1 | 2 | 3 | 4 | 2 | 3 | 2 | 4 | 2 | 2 | 2 | 3 | 4 | 3 |
| 79 | 1 | 2 | 1 | 2 | 3 | 2 | 1 | 1 | 1 | 2 | 1 | 1 | 1 | 1 | 1 | 2 | 1 | 1 | 2 | 3 | 2 |
| 80 | 2 | 2 | 2 | 2 | 2 | 2 | 1 | 4 | 2 | 2 | 2 | 3 | 2 | 2 | 2 | 3 | 3 | 2 | 2 | 3 | 2 |
| 81 | 2 | 2 | 2 | 2 | 4 | 2 | 2 | 2 | 2 | 2 | 2 | 2 | 2 | 2 | 4 | 2 | 2 | 1 | 3 | 1 | 1 |
| 82 | 2 | 2 | 2 | 1 | 5 | 1 | 2 | 3 | 3 | 3 | 2 | 2 | 2 | 2 | 2 | 2 | 2 | 1 | 3 | 2 | 3 |
| 83 | 1 | 4 | 1 | 2 | 2 | 1 | 1 | 1 | 1 | 1 | 1 | 1 | 1 | 1 | 1 | 1 | 1 | 1 | 1 | 2 | 1 |
| 84 | 1 | 4 | 1 | 1 | 4 | 2 | 1 | 2 | 1 | 3 | 1 | 1 | 2 | 1 | 1 | 1 | 1 | 1 | 2 | 2 | 2 |
| 85 | 1 | 3 | 1 | 2 | 2 | 1 | 1 | 1 | 1 | 1 | 2 | 2 | 2 | 1 | 2 | 2 | 3 | 2 | 4 | 3 | 2 |
| 86 | 1 | 5 | 5 | 5 | 5 | 5 | 5 | 5 | 5 | 2 | 5 | 5 | 2 | 2 | 2 | 5 | 5 | 5 | 5 | 5 | 1 |
| 87 | 2 | 2 | 1 | 3 | 3 | 1 | 1 | 1 | 1 | 1 | 1 | 1 | 3 | 1 | 3 | 2 | 1 | 1 | 3 | 3 | 1 |
| 88 | 2 | 2 | 1 | 3 | 3 | 1 | 1 | 1 | 1 | 1 | 1 | 1 | 3 | 1 | 3 | 2 | 1 | 1 | 3 | 3 | 1 |
| 89 | 1 | 4 | 4 | 1 | 4 | 1 | 1 | 1 | 4 | 4 | 5 | 1 | 4 | 1 | 5 | 2 | 1 | 2 | 4 | 5 | 2 |
| 90 | 1 | 2 | 2 | 1 | 3 | 2 | 1 | 3 | 1 | 1 | 2 | 2 | 2 | 1 | 2 | 2 | 1 | 1 | 1 | 2 | 2 |
| 91 | 1 | 4 | 2 | 2 | 1 | 2 | 1 | 2 | 1 | 2 | 3 | 1 | 1 | 1 | 2 | 1 | 2 | 1 | 2 | 3 | 2 |
| 92 | 2 | 2 | 2 | 2 | 2 | 1 | 2 | 3 | 2 | 2 | 3 | 1 | 1 | 1 | 2 | 2 | 1 | 2 | 2 | 2 | 2 |
| 93 | 1 | 4 | 2 | 1 | 2 | 1 | 2 | 1 | 1 | 2 | 2 | 1 | 2 | 1 | 2 | 2 | 1 | 2 | 2 | 3 | 1 |
| 94 | 2 | 3 | 1 | 1 | 3 | 1 | 4 | 2 | 4 | 2 | 4 | 4 | 2 | 1 | 3 | 2 | 3 | 1 | 1 | 1 | 2 |
| 95 | 2 | 2 | 3 | 2 | 2 | 1 | 2 | 3 | 2 | 2 | 3 | 1 | 1 | 1 | 2 | 2 | 1 | 2 | 2 | 2 | 2 |
| 96 | 1 | 1 | 1 | 1 | 2 | 1 | 1 | 1 | 1 | 1 | 1 | 1 | 3 | 1 | 3 | 1 | 1 | 1 | 1 | 3 | 1 |
| 97 | 2 | 2 | 3 | 2 | 2 | 1 | 2 | 3 | 2 | 2 | 3 | 1 | 1 | 1 | 2 | 1 | 2 | 1 | 2 | 2 | 2 |
| 98 | 2 | 2 | 3 | 2 | 2 | 1 | 2 | 3 | 2 | 2 | 3 | 1 | 1 | 1 | 2 | 2 | 1 | 2 | 1 | 2 | 2 |
| 99 | 2 | 2 | 1 | 2 | 2 | 2 | 2 | 1 | 1 | 2 | 2 | 3 | 2 | 2 | 3 | 3 | 2 | 2 | 2 | 2 | 2 |
| 100 | 2 | 3 | 2 | 1 | 2 | 2 | 1 | 2 | 1 | 2 | 3 | 2 | 3 | 1 | 2 | 1 | 2 | 1 | 3 | 3 | 1 |
| 101 | 1 | 4 | 1 | 1 | 2 | 1 | 1 | 1 | 1 | 2 | 1 | 1 | 1 | 1 | 2 | 2 | 2 | 4 | 1 | 1 | 1 |
| 102 | 2 | 1 | 1 | 1 | 4 | 2 | 1 | 4 | 2 | 1 | 2 | 2 | 1 | 1 | 4 | 2 | 2 | 1 | 4 | 4 | 3 |
| 103 | 1 | 2 | 1 | 1 | 2 | 1 | 1 | 2 | 5 | 2 | 1 | 1 | 1 | 1 | 2 | 1 | 1 | 1 | 2 | 2 | 1 |
| 104 | 1 | 2 | 1 | 1 | 1 | 1 | 1 | 1 | 3 | 1 | 1 | 1 | 1 | 1 | 1 | 1 | 1 | 1 | 1 | 1 | 1 |
| 105 | 2 | 1 | 1 | 1 | 1 | 1 | 1 | 1 | 1 | 1 | 1 | 1 | 1 | 1 | 4 | 1 | 1 | 1 | 1 | 1 | 1 |

## Appendix VI Learning Experience Questionnaire Data

### Table 6 Learning Experience Questionnaire Data

| NO. | EPR-1 | EPR-2 | EPR-3 | EPR-4 | EPR-5 | EPR-6 | EPR-7 | EPR-8 | EPR-9 | EPR-10 | EPR-11 | EPR-12 | EPR-13 | EPR-14 |
| --- | --- | --- | --- | --- | --- | --- | --- | --- | --- | --- | --- | --- | --- | --- |
| 1 | 1 | 1 | 4 | 4 | 4 | 4 | 4 | 4 | 4 | 4 | 1 | 5 | 4 | 4 |
| 2 | 4 | 4 | 4 | 2 | 2 | 4 | 1 | 1 | 3 | 2 | 3 | 4 | 4 | 4 |
| 3 | 3 | 3 | 3 | 3 | 3 | 3 | 3 | 3 | 2 | 3 | 5 | 3 | 3 | 3 |
| 4 | 3 | 2 | 4 | 3 | 3 | 2 | 2 | 3 | 3 | 4 | 4 | 4 | 2 | 3 |
| 5 | 2 | 2 | 2 | 3 | 2 | 2 | 2 | 1 | 2 | 1 | 2 | 2 | 2 | 3 |
| 6 | 2 | 2 | 3 | 3 | 3 | 2 | 3 | 1 | 3 | 2 | 3 | 3 | 2 | 2 |
| 7 | 4 | 2 | 4 | 2 | 2 | 4 | 2 | 1 | 2 | 2 | 4 | 4 | 1 | 1 |
| 8 | 2 | 1 | 2 | 4 | 4 | 2 | 2 | 1 | 5 | 1 | 5 | 5 | 1 | 1 |
| 9 | 3 | 3 | 3 | 3 | 3 | 4 | 3 | 1 | 4 | 1 | 3 | 3 | 1 | 1 |
| 10 | 2 | 3 | 2 | 4 | 3 | 3 | 2 | 2 | 3 | 3 | 3 | 3 | 2 | 3 |
| 11 | 2 | 2 | 2 | 4 | 2 | 2 | 4 | 2 | 4 | 1 | 2 | 4 | 3 | 4 |
| 12 | 2 | 3 | 2 | 3 | 3 | 2 | 2 | 2 | 2 | 3 | 2 | 2 | 3 | 3 |
| 13 | 3 | 4 | 4 | 2 | 3 | 2 | 2 | 4 | 4 | 1 | 4 | 3 | 2 | 3 |
| 14 | 5 | 5 | 4 | 4 | 2 | 2 | 5 | 5 | 5 | 4 | 2 | 2 | 2 | 2 |
| 15 | 2 | 3 | 2 | 2 | 3 | 2 | 3 | 1 | 3 | 2 | 2 | 3 | 2 | 2 |
| 16 | 2 | 3 | 2 | 2 | 3 | 3 | 2 | 1 | 4 | 3 | 3 | 3 | 2 | 3 |
| 17 | 1 | 2 | 2 | 2 | 3 | 2 | 2 | 2 | 4 | 2 | 3 | 3 | 2 | 1 |
| 18 | 2 | 2 | 3 | 2 | 2 | 2 | 1 | 2 | 3 | 3 | 3 | 2 | 2 | 2 |
| 19 | 2 | 2 | 2 | 2 | 3 | 2 | 4 | 2 | 2 | 2 | 2 | 2 | 3 | 3 |
| 20 | 1 | 3 | 4 | 3 | 3 | 2 | 4 | 4 | 1 | 2 | 4 | 2 | 4 | 2 |
| 21 | 1 | 4 | 2 | 2 | 4 | 1 | 3 | 2 | 5 | 1 | 4 | 2 | 3 | 3 |
| 22 | 2 | 2 | 3 | 5 | 2 | 5 | 4 | 2 | 2 | 4 | 2 | 2 | 1 | 2 |
| 23 | 3 | 2 | 2 | 2 | 2 | 2 | 3 | 1 | 1 | 1 | 2 | 1 | 3 | 3 |
| 24 | 2 | 2 | 2 | 1 | 2 | 2 | 2 | 1 | 2 | 2 | 2 | 2 | 3 | 3 |
| 25 | 2 | 2 | 2 | 2 | 3 | 1 | 2 | 2 | 2 | 2 | 2 | 2 | 4 | 1 |
| 26 | 2 | 3 | 2 | 2 | 2 | 3 | 2 | 2 | 2 | 2 | 2 | 2 | 2 | 2 |
| 27 | 4 | 2 | 2 | 2 | 2 | 2 | 2 | 2 | 4 | 2 | 2 | 2 | 3 | 3 |
| 28 | 3 | 2 | 3 | 3 | 3 | 2 | 4 | 2 | 2 | 2 | 3 | 2 | 2 | 3 |
| 29 | 2 | 3 | 4 | 4 | 2 | 2 | 2 | 2 | 3 | 2 | 3 | 2 | 2 | 3 |
| 30 | 2 | 1 | 1 | 1 | 2 | 2 | 2 | 2 | 2 | 4 | 1 | 1 | 4 | 3 |
| 31 | 2 | 2 | 2 | 2 | 2 | 2 | 2 | 2 | 2 | 1 | 2 | 2 | 3 | 3 |
| 32 | 2 | 1 | 3 | 4 | 2 | 1 | 1 | 1 | 2 | 2 | 2 | 2 | 3 | 3 |
| 33 | 1 | 1 | 2 | 2 | 3 | 3 | 2 | 1 | 3 | 2 | 3 | 3 | 2 | 3 |
| 34 | 3 | 2 | 2 | 2 | 2 | 2 | 3 | 2 | 2 | 2 | 2 | 2 | 3 | 3 |
| 35 | 2 | 3 | 3 | 3 | 4 | 3 | 1 | 3 | 2 | 1 | 4 | 3 | 2 | 1 |
| 36 | 2 | 3 | 3 | 3 | 4 | 3 | 1 | 3 | 2 | 1 | 4 | 3 | 2 | 1 |
| 37 | 2 | 2 | 2 | 2 | 2 | 3 | 3 | 3 | 3 | 4 | 2 | 2 | 2 | 3 |
| 38 | 2 | 2 | 4 | 4 | 3 | 3 | 3 | 2 | 2 | 2 | 2 | 2 | 2 | 2 |
| 39 | 2 | 2 | 3 | 2 | 4 | 3 | 4 | 1 | 2 | 2 | 3 | 3 | 3 | 4 |
| 40 | 3 | 4 | 3 | 4 | 2 | 3 | 2 | 3 | 2 | 3 | 2 | 2 | 3 | 3 |
| 41 | 2 | 3 | 3 | 3 | 3 | 3 | 3 | 2 | 2 | 3 | 3 | 2 | 3 | 4 |
| 42 | 2 | 2 | 1 | 1 | 2 | 1 | 3 | 2 | 2 | 2 | 4 | 2 | 2 | 3 |
| 43 | 3 | 3 | 4 | 2 | 3 | 3 | 2 | 2 | 1 | 1 | 2 | 2 | 3 | 3 |
| 44 | 4 | 4 | 3 | 2 | 4 | 4 | 1 | 1 | 2 | 1 | 4 | 1 | 1 | 3 |
| 45 | 2 | 2 | 3 | 3 | 3 | 2 | 3 | 3 | 2 | 2 | 3 | 2 | 3 | 3 |
| 46 | 2 | 2 | 1 | 3 | 2 | 2 | 5 | 4 | 2 | 4 | 2 | 2 | 3 | 3 |
| 47 | 3 | 4 | 4 | 4 | 3 | 2 | 2 | 2 | 4 | 4 | 3 | 2 | 2 | 3 |
| 48 | 2 | 4 | 1 | 1 | 1 | 1 | 1 | 2 | 1 | 1 | 1 | 1 | 1 | 5 |
| 49 | 1 | 3 | 3 | 1 | 2 | 2 | 1 | 3 | 5 | 3 | 1 | 1 | 1 | 2 |
| 50 | 2 | 2 | 2 | 2 | 3 | 2 | 2 | 1 | 2 | 2 | 2 | 3 | 3 | 3 |
| 51 | 2 | 2 | 2 | 2 | 2 | 2 | 2 | 4 | 2 | 4 | 2 | 2 | 3 | 3 |
| 52 | 2 | 2 | 2 | 3 | 2 | 4 | 3 | 3 | 2 | 2 | 3 | 2 | 3 | 3 |
| 53 | 1 | 1 | 2 | 4 | 4 | 3 | 3 | 4 | 2 | 4 | 2 | 2 | 3 | 3 |
| 54 | 1 | 1 | 1 | 1 | 2 | 1 | 2 | 1 | 1 | 1 | 1 | 1 | 1 | 1 |
| 55 | 1 | 1 | 1 | 1 | 1 | 1 | 1 | 1 | 1 | 4 | 1 | 1 | 3 | 3 |
| 56 | 1 | 1 | 1 | 3 | 3 | 1 | 2 | 2 | 2 | 2 | 2 | 2 | 1 | 2 |
| 57 | 2 | 2 | 2 | 1 | 1 | 2 | 2 | 4 | 1 | 4 | 1 | 2 | 3 | 3 |
| 58 | 4 | 3 | 2 | 2 | 2 | 2 | 2 | 2 | 2 | 2 | 2 | 2 | 2 | 2 |
| 59 | 1 | 1 | 3 | 4 | 2 | 2 | 3 | 2 | 3 | 4 | 2 | 3 | 3 | 3 |
| 60 | 1 | 1 | 2 | 2 | 3 | 3 | 2 | 1 | 2 | 2 | 3 | 3 | 2 | 3 |
| 61 | 1 | 1 | 3 | 2 | 2 | 2 | 2 | 1 | 2 | 1 | 4 | 4 | 3 | 3 |
| 62 | 2 | 2 | 2 | 3 | 2 | 1 | 3 | 3 | 2 | 1 | 3 | 2 | 3 | 3 |
| 63 | 1 | 2 | 2 | 3 | 2 | 2 | 1 | 5 | 2 | 5 | 1 | 1 | 3 | 3 |
| 64 | 3 | 3 | 2 | 3 | 3 | 2 | 2 | 2 | 2 | 2 | 3 | 4 | 1 | 1 |
| 65 | 1 | 1 | 2 | 4 | 5 | 1 | 5 | 1 | 2 | 2 | 4 | 1 | 1 | 3 |
| 66 | 2 | 2 | 1 | 2 | 2 | 1 | 1 | 1 | 1 | 2 | 1 | 2 | 3 | 2 |
| 67 | 1 | 1 | 2 | 2 | 4 | 2 | 1 | 1 | 1 | 1 | 3 | 4 | 1 | 2 |
| 68 | 2 | 2 | 3 | 2 | 3 | 2 | 1 | 1 | 2 | 1 | 3 | 3 | 2 | 2 |
| 69 | 2 | 2 | 2 | 5 | 3 | 1 | 2 | 3 | 3 | 1 | 2 | 2 | 2 | 3 |
| 70 | 1 | 1 | 4 | 2 | 5 | 2 | 2 | 1 | 5 | 2 | 1 | 2 | 3 | 3 |
| 71 | 1 | 1 | 2 | 2 | 4 | 4 | 2 | 3 | 2 | 2 | 2 | 3 | 3 | 3 |
| 72 | 2 | 1 | 2 | 2 | 4 | 4 | 4 | 1 | 4 | 2 | 2 | 2 | 3 | 2 |
| 73 | 2 | 2 | 2 | 3 | 2 | 3 | 3 | 2 | 3 | 4 | 2 | 2 | 3 | 3 |
| 74 | 1 | 1 | 1 | 1 | 1 | 1 | 1 | 1 | 1 | 1 | 1 | 1 | 2 | 4 |
| 75 | 1 | 2 | 2 | 2 | 2 | 1 | 1 | 1 | 5 | 4 | 3 | 3 | 1 | 1 |
| 76 | 2 | 2 | 2 | 2 | 2 | 4 | 1 | 1 | 2 | 2 | 2 | 2 | 3 | 2 |
| 77 | 2 | 2 | 2 | 2 | 2 | 2 | 3 | 2 | 2 | 2 | 2 | 1 | 3 | 3 |
| 78 | 2 | 2 | 2 | 4 | 2 | 2 | 3 | 4 | 2 | 1 | 2 | 1 | 3 | 3 |
| 79 | 2 | 1 | 4 | 4 | 5 | 2 | 1 | 3 | 1 | 1 | 3 | 1 | 3 | 3 |
| 80 | 2 | 2 | 3 | 3 | 3 | 2 | 3 | 2 | 3 | 3 | 2 | 3 | 2 | 2 |
| 81 | 2 | 1 | 1 | 2 | 2 | 2 | 1 | 5 | 2 | 3 | 5 | 3 | 4 | 2 |
| 82 | 3 | 2 | 2 | 2 | 2 | 2 | 3 | 2 | 2 | 3 | 5 | 4 | 3 | 3 |
| 83 | 2 | 4 | 3 | 2 | 1 | 1 | 1 | 2 | 1 | 3 | 1 | 1 | 2 | 2 |
| 84 | 1 | 2 | 3 | 2 | 3 | 2 | 4 | 2 | 3 | 1 | 1 | 2 | 3 | 3 |
| 85 | 2 | 3 | 3 | 4 | 3 | 3 | 2 | 2 | 3 | 1 | 3 | 3 | 3 | 3 |
| 86 | 2 | 3 | 5 | 5 | 5 | 5 | 2 | 4 | 2 | 4 | 2 | 4 | 5 | 2 |
| 87 | 1 | 1 | 2 | 1 | 2 | 3 | 1 | 1 | 2 | 4 | 2 | 2 | 3 | 4 |
| 88 | 1 | 1 | 2 | 1 | 2 | 3 | 1 | 1 | 2 | 4 | 2 | 2 | 3 | 4 |
| 89 | 1 | 2 | 2 | 2 | 3 | 1 | 4 | 1 | 2 | 2 | 2 | 3 | 3 | 2 |
| 90 | 2 | 2 | 2 | 4 | 3 | 2 | 1 | 3 | 1 | 2 | 2 | 2 | 1 | 3 |
| 91 | 2 | 2 | 1 | 3 | 2 | 1 | 2 | 3 | 2 | 4 | 2 | 2 | 3 | 2 |
| 92 | 3 | 4 | 2 | 1 | 4 | 1 | 2 | 2 | 2 | 2 | 1 | 3 | 3 | 2 |
| 93 | 1 | 2 | 2 | 2 | 2 | 2 | 2 | 1 | 2 | 3 | 2 | 2 | 3 | 1 |
| 94 | 2 | 2 | 4 | 2 | 2 | 2 | 1 | 3 | 1 | 1 | 2 | 2 | 2 | 3 |
| 95 | 3 | 4 | 2 | 1 | 1 | 1 | 2 | 2 | 2 | 2 | 1 | 3 | 3 | 2 |
| 96 | 2 | 2 | 1 | 1 | 2 | 2 | 2 | 1 | 2 | 4 | 3 | 1 | 3 | 3 |
| 97 | 3 | 4 | 2 | 1 | 3 | 1 | 2 | 2 | 2 | 1 | 1 | 3 | 3 | 2 |
| 98 | 3 | 4 | 2 | 1 | 3 | 1 | 2 | 2 | 2 | 2 | 1 | 3 | 3 | 2 |
| 99 | 2 | 2 | 2 | 2 | 2 | 4 | 4 | 2 | 4 | 2 | 4 | 2 | 1 | 3 |
| 100 | 1 | 1 | 3 | 1 | 2 | 1 | 3 | 1 | 2 | 1 | 2 | 2 | 3 | 2 |
| 101 | 1 | 1 | 1 | 1 | 1 | 1 | 2 | 1 | 2 | 2 | 1 | 1 | 2 | 1 |
| 102 | 1 | 2 | 3 | 3 | 4 | 1 | 1 | 4 | 4 | 1 | 3 | 1 | 2 | 3 |
| 103 | 1 | 1 | 1 | 5 | 1 | 3 | 1 | 1 | 5 | 2 | 2 | 1 | 2 | 4 |
| 104 | 1 | 1 | 1 | 2 | 2 | 1 | 1 | 1 | 1 | 1 | 1 | 1 | 2 | 3 |
| 105 | 1 | 1 | 1 | 1 | 1 | 1 | 1 | 1 | 1 | 1 | 1 | 3 | 1 | 1 |

## Appendix VII Leaning Outcomes Questionnaire Data

### Table 7 Leaning Outcomes Questionnaire Data

| NO. | OL-1 | OL-2 | OL-3 | OL-4 | OL-5 | OL-6 | OL-7 | OL-8 | OL-9 | OL-10 | OL-11 | OL-12 | OL-13 | OL-14 |
| --- | --- | --- | --- | --- | --- | --- | --- | --- | --- | --- | --- | --- | --- | --- |
| 1 | 4 | 4 | 4 | 4 | 4 | 4 | 4 | 4 | 4 | 4 | 4 | 4 | 4 | 4 |
| 2 | 5 | 5 | 4 | 4 | 1 | 1 | 1 | 5 | 1 | 5 | 1 | 4 | 5 | 5 |
| 3 | 5 | 2 | 2 | 3 | 2 | 2 | 3 | 5 | 4 | 5 | 3 | 3 | 4 | 4 |
| 4 | 2 | 4 | 3 | 4 | 4 | 2 | 3 | 4 | 2 | 3 | 3 | 4 | 2 | 4 |
| 5 | 2 | 2 | 3 | 4 | 5 | 3 | 3 | 2 | 1 | 4 | 3 | 4 | 5 | 2 |
| 6 | 5 | 2 | 2 | 2 | 3 | 3 | 2 | 4 | 2 | 3 | 2 | 3 | 4 | 5 |
| 7 | 2 | 2 | 4 | 4 | 2 | 2 | 2 | 4 | 2 | 4 | 4 | 2 | 4 | 4 |
| 8 | 5 | 1 | 5 | 1 | 1 | 2 | 2 | 5 | 2 | 5 | 3 | 2 | 2 | 5 |
| 9 | 4 | 2 | 3 | 4 | 3 | 2 | 2 | 2 | 2 | 4 | 2 | 2 | 3 | 5 |
| 10 | 3 | 2 | 3 | 4 | 3 | 2 | 3 | 3 | 2 | 3 | 2 | 4 | 3 | 2 |
| 11 | 2 | 2 | 3 | 3 | 4 | 3 | 2 | 2 | 1 | 4 | 2 | 2 | 4 | 5 |
| 12 | 2 | 3 | 3 | 3 | 3 | 2 | 3 | 3 | 2 | 2 | 3 | 4 | 3 | 2 |
| 13 | 3 | 2 | 3 | 2 | 2 | 1 | 1 | 4 | 3 | 5 | 2 | 3 | 4 | 3 |
| 14 | 3 | 1 | 2 | 2 | 2 | 3 | 3 | 3 | 1 | 4 | 4 | 3 | 3 | 4 |
| 15 | 2 | 2 | 3 | 2 | 4 | 2 | 2 | 3 | 2 | 3 | 2 | 4 | 3 | 3 |
| 16 | 2 | 3 | 3 | 2 | 4 | 2 | 2 | 2 | 2 | 4 | 2 | 2 | 2 | 5 |
| 17 | 1 | 3 | 3 | 5 | 5 | 1 | 1 | 3 | 1 | 1 | 1 | 5 | 3 | 3 |
| 18 | 2 | 2 | 4 | 4 | 4 | 1 | 2 | 1 | 3 | 4 | 2 | 4 | 2 | 1 |
| 19 | 2 | 3 | 2 | 3 | 2 | 3 | 2 | 2 | 2 | 3 | 2 | 3 | 4 | 3 |
| 20 | 3 | 2 | 4 | 2 | 4 | 1 |  | 2 | 1 | 5 | 3 | 4 | 2 | 1 |
| 21 | 2 | 4 | 3 | 3 | 4 | 1 | 1 | 2 | 2 | 2 | 1 | 4 | 5 | 2 |
| 22 | 2 | 2 | 2 | 4 | 2 | 4 | 2 | 2 | 2 | 4 | 4 | 2 | 2 | 2 |
| 23 | 2 | 3 | 5 | 3 | 2 | 2 | 2 | 2 | 2 | 2 | 2 | 3 | 3 | 2 |
| 24 | 1 | 3 | 3 | 4 | 4 | 2 | 2 | 2 | 1 | 2 | 1 | 5 | 3 | 2 |
| 25 | 1 | 4 | 2 | 4 | 3 | 3 | 2 | 2 | 2 | 2 | 2 | 4 | 2 | 2 |
| 26 | 3 | 2 | 2 | 4 | 3 | 2 | 2 | 2 | 3 | 2 | 3 | 3 | 2 | 2 |
| 27 | 2 | 2 | 2 | 4 | 4 | 2 | 2 | 2 | 2 | 2 | 2 | 4 | 2 | 3 |
| 28 | 2 | 3 | 3 | 3 | 3 | 2 | 2 | 3 | 2 | 2 | 2 | 3 | 3 | 2 |
| 29 | 2 | 2 | 2 | 2 | 2 | 2 | 2 | 2 | 2 | 4 | 2 | 4 | 4 | 3 |
| 30 | 1 | 4 | 1 | 2 | 4 | 3 | 2 | 2 | 2 | 2 | 2 | 5 | 3 | 1 |
| 31 | 2 | 2 | 2 | 2 | 4 | 2 | 2 | 2 | 1 | 2 | 2 | 4 | 4 | 3 |
| 32 | 2 | 4 | 1 | 3 | 4 | 2 | 2 | 2 | 2 | 2 | 2 | 4 | 2 | 2 |
| 33 | 3 | 3 | 3 | 2 | 2 | 2 | 2 | 2 | 1 | 4 | 2 | 1 | 4 | 3 |
| 34 | 2 | 2 | 3 | 4 | 2 | 1 | 2 | 2 | 2 | 2 | 2 | 4 | 3 | 3 |
| 35 | 1 | 4 | 3 | 3 | 2 | 2 | 2 | 3 | 3 | 3 | 1 | 1 | 3 | 3 |
| 36 | 1 | 4 | 3 | 3 | 2 | 2 | 2 | 3 | 3 | 3 | 1 | 1 | 3 | 3 |
| 37 | 2 | 2 | 4 | 2 | 3 | 2 | 3 | 2 | 2 | 3 | 2 | 3 | 2 | 2 |
| 38 | 2 | 3 | 4 | 4 | 3 | 1 | 2 | 2 | 1 | 2 | 2 | 3 | 3 | 2 |
| 39 | 4 | 2 | 2 | 2 | 2 | 2 | 1 | 4 | 1 | 4 | 1 | 2 | 3 | 4 |
| 40 | 1 | 3 | 2 | 4 | 4 | 2 | 2 | 1 | 2 | 2 | 3 | 5 | 2 | 1 |
| 41 | 3 | 2 | 2 | 3 | 3 | 2 | 2 | 2 | 2 | 2 | 3 | 3 | 2 | 3 |
| 42 | 2 | 4 | 5 | 4 | 2 | 2 | 2 | 2 | 2 | 2 | 2 | 2 | 1 | 1 |
| 43 | 2 | 4 | 2 | 4 | 4 | 1 | 2 | 3 | 2 | 2 | 2 | 2 | 1 | 2 |
| 44 | 1 | 2 | 2 | 2 | 1 | 1 | 1 | 4 | 1 | 4 | 1 | 4 | 4 | 5 |
| 45 | 2 | 2 | 3 | 3 | 2 | 1 | 2 | 4 | 2 | 2 | 2 | 4 | 2 | 2 |
| 46 | 1 | 3 | 3 | 4 | 5 | 1 | 3 | 2 | 1 | 1 | 2 | 4 | 2 | 1 |
| 47 | 3 | 2 | 4 | 2 | 2 | 2 | 1 | 3 | 2 | 3 | 3 | 2 | 2 | 2 |
| 48 | 1 | 1 | 1 | 1 | 1 | 1 | 3 | 1 | 4 | 2 | 5 | 4 | 3 | 4 |
| 49 | 2 | 4 | 1 | 5 | 3 | 1 | 2 | 1 | 1 | 2 | 3 | 2 | 3 | 2 |
| 50 | 2 | 4 | 2 | 4 | 4 | 1 | 1 | 2 | 2 | 2 | 1 | 4 | 2 | 1 |
| 51 | 2 | 4 | 2 | 2 | 2 | 2 | 2 | 3 | 2 | 2 | 2 | 3 | 2 | 2 |
| 52 | 2 | 2 | 3 | 3 | 3 | 1 | 1 | 2 | 2 | 2 | 2 | 3 | 3 | 3 |
| 53 | 3 | 1 | 2 | 3 | 3 | 2 | 1 | 2 | 3 | 1 | 2 | 3 | 3 | 3 |
| 54 | 2 | 2 | 4 | 5 | 5 | 1 | 1 | 2 | 1 | 1 | 1 | 4 | 1 | 1 |
| 55 | 1 | 4 | 4 | 4 | 5 | 1 | 1 | 1 | 1 | 1 | 1 | 5 | 1 | 1 |
| 56 | 3 | 3 | 3 | 3 | 3 | 1 | 2 | 1 | 1 | 2 | 1 | 1 | 2 | 5 |
| 57 | 1 | 2 | 2 | 4 | 5 | 1 | 2 | 3 | 1 | 1 | 1 | 4 | 2 | 2 |
| 58 | 2 | 2 | 2 | 2 | 2 | 2 | 2 | 2 | 2 | 2 | 2 | 2 | 4 | 3 |
| 59 | 3 | 1 | 2 | 3 | 3 | 2 | 1 | 3 | 2 | 3 | 2 | 2 | 2 | 2 |
| 60 | 3 | 3 | 3 | 2 | 2 | 2 | 2 | 2 | 2 | 2 | 2 | 2 | 2 | 1 |
| 61 | 2 | 2 | 2 | 2 | 2 | 1 | 2 | 3 | 2 | 3 | 2 | 1 | 3 | 3 |
| 62 | 1 | 2 | 2 | 4 | 5 | 1 | 1 | 2 | 2 | 2 | 2 | 4 | 1 | 1 |
| 63 | 2 | 3 | 2 | 3 | 4 | 2 | 1 | 3 | 1 | 1 | 2 | 3 | 2 | 1 |
| 64 | 1 | 1 | 2 | 2 | 1 | 1 | 1 | 3 | 2 | 3 | 2 | 3 | 4 | 4 |
| 65 | 1 | 4 | 2 | 4 | 5 | 1 | 1 | 1 | 1 | 1 | 1 | 5 | 1 | 2 |
| 66 | 1 | 4 | 2 | 4 | 2 | 1 | 2 | 2 | 2 | 1 | 4 | 2 | 1 | 1 |
| 67 | 4 | 2 | 2 | 2 | 1 | 1 | 2 | 3 | 1 | 1 | 2 | 3 | 2 | 2 |
| 68 | 2 | 3 | 2 | 4 | 2 | 1 | 2 | 2 | 2 | 2 | 2 | 1 | 2 | 2 |
| 69 | 2 | 1 | 2 | 4 | 1 | 1 | 1 | 2 | 2 | 2 | 3 | 3 | 3 | 2 |
| 70 | 2 | 2 | 2 | 2 | 1 | 1 | 2 | 2 | 2 | 3 | 2 | 4 | 2 | 2 |
| 71 | 1 | 4 | 2 | 4 | 3 | 1 | 2 | 1 | 1 | 1 | 2 | 4 | 2 | 1 |
| 72 | 2 | 2 | 2 | 4 | 2 | 1 | 2 | 2 | 2 | 1 | 4 | 2 | 2 | 1 |
| 73 | 2 | 1 | 2 | 2 | 3 | 2 | 2 | 2 | 1 | 2 | 2 | 3 | 2 | 3 |
| 74 | 2 | 2 | 2 | 2 | 2 | 2 | 2 | 2 | 2 | 2 | 2 | 2 | 2 | 2 |
| 75 | 1 | 1 | 1 | 2 | 3 | 1 | 1 | 4 | 1 | 2 | 1 | 5 | 2 | 3 |
| 76 | 1 | 1 | 2 | 4 | 4 | 1 | 1 | 2 | 1 | 1 | 4 | 4 | 1 | 1 |
| 77 | 2 | 1 | 1 | 2 | 4 | 1 | 1 | 2 | 1 | 2 | 3 | 4 | 2 | 2 |
| 78 | 2 | 1 | 3 | 2 | 4 | 1 | 1 | 2 | 1 | 2 | 1 | 5 | 2 | 1 |
| 79 | 1 | 4 | 2 | 2 | 4 | 1 | 1 | 3 | 1 | 1 | 2 | 4 | 1 | 1 |
| 80 | 2 | 2 | 2 | 2 | 2 | 2 | 2 | 2 | 2 | 2 | 2 | 2 | 2 | 2 |
| 81 | 2 | 1 | 4 | 1 | 4 | 1 | 1 | 3 | 1 | 1 | 3 | 3 | 2 | 1 |
| 82 | 2 | 2 | 2 | 2 | 2 | 1 | 2 | 2 | 2 | 2 | 2 | 2 | 3 | 2 |
| 83 | 1 | 1 | 3 | 3 | 3 | 1 | 1 | 4 | 1 | 1 | 1 | 4 | 1 | 2 |
| 84 | 2 | 2 | 2 | 2 | 3 | 1 | 2 | 4 | 1 | 2 | 2 | 2 | 1 | 1 |
| 85 | 2 | 3 | 2 | 4 | 3 | 1 | 1 | 1 | 2 | 2 | 2 | 1 | 1 | 2 |
| 86 | 1 | 2 | 2 | 2 | 2 | 2 | 2 | 2 | 2 | 2 | 2 | 2 | 2 | 2 |
| 87 | 1 | 1 | 2 | 3 | 4 | 2 | 1 | 1 | 1 | 2 | 2 | 3 | 1 | 2 |
| 88 | 1 | 1 | 2 | 3 | 4 | 2 | 1 | 1 | 1 | 2 | 2 | 3 | 1 | 2 |
| 89 | 1 | 1 | 2 | 3 | 4 | 1 | 1 | 1 | 1 | 2 | 1 | 4 | 2 | 2 |
| 90 | 2 | 2 | 2 | 2 | 2 | 2 | 2 | 2 | 1 | 2 | 2 | 1 | 2 | 2 |
| 91 | 2 | 1 | 2 | 3 | 3 | 1 | 2 | 2 | 1 | 1 | 1 | 2 | 3 | 2 |
| 92 | 2 | 2 | 1 | 1 | 1 | 2 | 2 | 1 | 2 | 2 | 1 | 2 | 2 | 5 |
| 93 | 1 | 2 | 1 | 3 | 2 | 1 | 2 | 3 | 1 | 1 | 2 | 1 | 3 | 2 |
| 94 | 3 | 1 | 1 | 2 | 1 | 1 | 1 | 3 | 1 | 3 | 1 | 1 | 3 | 3 |
| 95 | 2 | 2 | 1 | 1 | 1 | 2 | 2 | 1 | 1 | 2 | 2 | 1 | 2 | 5 |
| 96 | 2 | 2 | 2 | 3 | 3 | 1 | 1 | 2 | 1 | 2 | 2 | 2 | 1 | 1 |
| 97 | 2 | 2 | 1 | 1 | 1 | 2 | 2 | 1 | 1 | 2 | 2 | 1 | 2 | 5 |
| 98 | 2 | 2 | 1 | 1 | 1 | 2 | 2 | 1 | 1 | 2 | 2 | 1 | 2 | 5 |
| 99 | 1 | 2 | 2 | 3 | 2 | 2 | 2 | 2 | 2 | 2 | 1 | 2 | 1 | 1 |
| 100 | 2 | 1 | 1 | 3 | 3 | 1 | 1 | 1 | 1 | 2 | 2 | 2 | 1 | 1 |
| 101 | 1 | 1 | 1 | 1 | 1 | 1 | 1 | 1 | 1 | 1 | 1 | 2 | 2 | 5 |
| 102 | 2 | 2 | 1 | 2 | 2 | 1 | 1 | 1 | 1 | 2 | 1 | 2 | 1 | 1 |
| 103 | 1 | 2 | 2 | 2 | 3 | 1 | 1 | 1 | 1 | 1 | 1 | 1 | 1 | 1 |
| 104 | 1 | 1 | 1 | 1 | 1 | 1 | 1 | 2 | 2 | 1 | 2 | 1 | 1 | 1 |
| 105 | 1 | 1 | 1 | 1 | 1 | 1 | 1 | 2 | 1 | 1 | 1 | 1 | 1 | 1 |

## Appendix VIII Factor Tests

### Table 8 Total Variance of Leaning Motivation

| Item | Initial eigenvalue | | | Retrieve sum of squares loading | | | Cyclic sum of squares loading | | |
| --- | --- | --- | --- | --- | --- | --- | --- | --- | --- |
|  | Total | Variant % | Accumulative % | Total | Variant % | Accumulative % | Total | Variant % | Accumulative % |
| 1 | 6.182 | 30.911 | 30.911 | 6.182 | 30.911 | 30.911 | 4.300 | 21.499 | 21.499 |
| 2 | 2.261 | 11.307 | 42.218 | 2.261 | 11.307 | 42.218 | 2.858 | 14.290 | 35.789 |
| 3 | 1.303 | 6.514 | 48.732 | 1.303 | 6.514 | 48.732 | 1.829 | 9.147 | 44.936 |
| 4 | 1.230 | 6.150 | 54.882 | 1.230 | 6.150 | 54.882 | 1.574 | 7.871 | 52.807 |
| 5 | 1.015 | 5.074 | 59.956 | 1.015 | 5.074 | 59.956 | 1.430 | 7.148 | 59.956 |
| 6 | .993 | 4.965 | 64.920 |  |  |  |  |  |  |
| 7 | .870 | 4.349 | 69.269 |  |  |  |  |  |  |
| 8 | .777 | 3.884 | 73.153 |  |  |  |  |  |  |
| 9 | .751 | 3.755 | 76.908 |  |  |  |  |  |  |
| 10 | .744 | 3.721 | 80.629 |  |  |  |  |  |  |
| 11 | .625 | 3.124 | 83.753 |  |  |  |  |  |  |
| 12 | .542 | 2.712 | 86.465 |  |  |  |  |  |  |
| 13 | .501 | 2.507 | 88.972 |  |  |  |  |  |  |
| 14 | .470 | 2.348 | 91.320 |  |  |  |  |  |  |
| 15 | .425 | 2.125 | 93.445 |  |  |  |  |  |  |
| 16 | .349 | 1.744 | 95.189 |  |  |  |  |  |  |
| 17 | .288 | 1.438 | 96.627 |  |  |  |  |  |  |
| 18 | .251 | 1.257 | 97.884 |  |  |  |  |  |  |
| 19 | .241 | 1.204 | 99.087 |  |  |  |  |  |  |
| 20 | .183 | .913 | 100.000 |  |  |  |  |  |  |

*Note*. Extraction method: main component analysis. Rotation method: the largest variation method with Kaiser regularization.

### Table 9 Rotating Element Matrix of Leaning Motivation

| Item | Component | | | | |
| --- | --- | --- | --- | --- | --- |
|  | 1 | 2 | 3 | 4 | 5 |
| M-1 | .289 | -.291 | -.118 | .419 | .615 |
| M-2 | -.017 | .018 | .768 | .160 | -.154 |
| M-3 | .274 | .297 | .627 | .007 | .192 |
| M-4 | .722 | -.046 | .084 | -.009 | .293 |
| M-5 | .096 | .698 | .154 | .065 | .001 |
| M-6 | .569 | .307 | .015 | .266 | -.049 |
| M-7 | .730 | -.059 | .283 | .098 | .196 |
| M-8 | .461 | -.058 | .328 | .385 | .081 |
| M-10 | .250 | .407 | .098 | .568 | .133 |
| M-11 | .185 | .196 | .555 | .057 | .372 |
| M-12 | .822 | .089 | -.010 | .163 | .070 |
| M-13 | .212 | .762 | .026 | .090 | .124 |
| M-14 | .527 | .164 | .066 | .354 | .250 |
| M-15 | .083 | .347 | .153 | -.060 | .778 |
| M-16 | .779 | .151 | .101 | -.097 | .009 |
| M-17 | .716 | .293 | .022 | .147 | -.047 |
| M-18 | .539 | .287 | .336 | -.255 | .041 |
| M-19 | .013 | .725 | .006 | .211 | .043 |
| M-20 | .168 | .577 | .351 | .099 | .081 |
| M-21 | .003 | .324 | .148 | .707 | -.030 |

*Note.* Rotation method: the largest variation method with Kaiser regularization. M= Motivation.

### Table 10 Total Variance of Leaning Experience

| Item | Initial eigenvalue | | | Retrieve sum of squares loading | | | Cyclic sum of squares loading | | |
| --- | --- | --- | --- | --- | --- | --- | --- | --- | --- |
|  | Total | Variant % | Accumulative % | Total | Variant % | Accumulative % | Total | Variant % | Accumulative % |
| 1 | 3.033 | 27.575 | 27.575 | 3.033 | 27.575 | 27.575 | 2.710 | 24.640 | 24.640 |
| 2 | 1.556 | 14.145 | 41.720 | 1.556 | 14.145 | 41.720 | 1.834 | 16.675 | 41.315 |
| 3 | 1.314 | 11.949 | 53.669 | 1.314 | 11.949 | 53.669 | 1.359 | 12.354 | 53.669 |
| 4 | .909 | 8.265 | 61.935 |  |  |  |  |  |  |
| 5 | .875 | 7.952 | 69.887 |  |  |  |  |  |  |
| 6 | .836 | 7.602 | 77.489 |  |  |  |  |  |  |
| 7 | .716 | 6.509 | 83.998 |  |  |  |  |  |  |
| 8 | .533 | 4.845 | 88.843 |  |  |  |  |  |  |
| 9 | .466 | 4.239 | 93.082 |  |  |  |  |  |  |
| 10 | .442 | 4.022 | 97.104 |  |  |  |  |  |  |
| 11 | .319 | 2.896 | 100.000 |  |  |  |  |  |  |

*Note*. Extraction method: main component analysis.

### Table 11 Rotating Element Matrix of Leaning Experience

| Item | Component | | |
| --- | --- | --- | --- |
|  | 1 | 2 | 3 |
| EPR-1 | .041 | .854 | -.060 |
| EPR-2 | .078 | .879 | .059 |
| EPR-3 | .623 | .363 | .183 |
| EPR-4 | .617 | .004 | .473 |
| EPR-5 | .705 | -.019 | -.028 |
| EPR-6 | .576 | .008 | .179 |
| EPR-8 | .177 | .387 | .590 |
| EPR-9 | .486 | .038 | -.076 |
| EPR-11 | .695 | .112 | -.130 |
| EPR-12 | .591 | .182 | -.516 |
| EPR-14 | -.067 | -.063 | .652 |

*Note*. Rotation method: the largest variation method with Kaiser regularization. EPR= Experience.

### Table 12 Total Variance of Leaning Outcomes

| Item | Initial eigenvalue | | | Retrieve sum of squares loading | | | Cyclic sum of squares loading | | |
| --- | --- | --- | --- | --- | --- | --- | --- | --- | --- |
|  | Total | Variant % | Accumulative % | Total | Variant % | Accumulative % | Total | Variant % | Accumulative % |
| 1 | 3.556 | 39.515 | 39.515 | 3.556 | 39.515 | 39.515 | 2.831 | 31.459 | 31.459 |
| 2 | 1.535 | 17.051 | 56.566 | 1.535 | 17.051 | 56.566 | 2.260 | 25.107 | 56.566 |
| 3 | .929 | 10.320 | 66.886 |  |  |  |  |  |  |
| 4 | .658 | 7.314 | 74.200 |  |  |  |  |  |  |
| 5 | .577 | 6.414 | 80.614 |  |  |  |  |  |  |
| 6 | .558 | 6.198 | 86.812 |  |  |  |  |  |  |
| 7 | .468 | 5.202 | 92.015 |  |  |  |  |  |  |
| 8 | .414 | 4.597 | 96.611 |  |  |  |  |  |  |
| 9 | .305 | 3.389 | 100.000 |  |  |  |  |  |  |

*Note*. Extraction method: main component analysis.

### Table 13 Rotating Element Matrix of Leaning Outcomes

| Item | Component | |
| --- | --- | --- |
|  | 1 | 2 |
| LO-1 | .727 | .182 |
| LO-6 | .302 | .646 |
| LO-7 | .156 | .795 |
| LO-8 | .662 | .011 |
| LO-9 | .190 | .662 |
| LO-10 | .800 | .299 |
| LO-11 | -.057 | .780 |
| LO-13 | .724 | .194 |
| LO-14 | .739 | .067 |

*Note*. Rotation method: the largest variation method with Kaiser regularization. LO=Leaning Outcomes.

## Appendix IX Regression Analysis Data

### Table 14 Model 1 Regression Analysis Data

| CORRELATION MATRIX | | | | | | | | | |
| --- | --- | --- | --- | --- | --- | --- | --- | --- | --- |
|  | | Gender | | Ages | | Language | | Non-Cognitive Learning Outcomes | |
| Gender | | 1.000 | | 0.072 | | 0.097 | | 0.076 | |
| Ages | | 0.072 | | 1.000 | | -0.135 | | -0.177 | |
| Language | | 0.097 | | -0.135 | | 1.000 | | 0.142 | |
| Non-Cognitive Learning Outcomes | | 0.076 | | -0.177 | | 0.142 | | 1.000 | |
| Determinant | | 0.96511330 | | | | | | | |
| MODEL FIT | | | | | | | | | |
| R-square | | Adjusted R-square | | F Value | | One-Tailed Probability | | | |
| 0.051 | | 0.014 | | 1.807 | | 0.172 | | | |
| REGRESSION COEFFICIENTS | | | | | | | | | |
| Independent | Un-stdized Coefficient | | St'dized Coefficient | | Proportion As Large | | Proportion As Small | | Proportion As Extreme |
| Intercept | -0.093866 | | 0.000000 | | 1.000 | | 0.000 | | 1.000 |
| Gender | 0.164358 | | 0.077280 | | 0.309 | | 0.691 | | 0.406 |
| Ages | -0.259728 | | -0.167107 | | 0.979 | | 0.021 | | 0.100 |
| Language | 0.332800 | | 0.112332 | | 0.011 | | 0.989 | | 0.120 |

*Note*. All probabilities based on randomization tests.

### Table 15 Model 2 Regression Analysis Data

| MODEL FIT | | | | | | | | |
| --- | --- | --- | --- | --- | --- | --- | --- | --- |
| R-square | Adjusted R-square | | | F Value | | | One-Tailed Probability | |
| 0.260 | 0.190 | | | 4.165 | | | 0.037 | |
| REGRESSION COEFFICIENTS | | | | | | | | |
| Independent | | Un-stdized Coefficient | St'dized Coefficient | | Proportion As Large | Proportion As Small | | Proportion As Extreme |
| Intercept | | 0.601427 | 0.000000 | | 1.000 | 0.000 | | 1.000 |
| Gender | | 0.089203 | 0.041943 | | 0.363 | 0.637 | | 0.886 |
| Ages | | 0.228635 | 0.147103 | | 0.901 | 0.099 | | 0.198 |
| Language | | 0.029105 | 0.009570 | | 0.323 | 0.677 | | 0.695 |
| Social Motivation (A) | | 0.258128 | 0.258140 | | 0.019 | 0.981 | | 0.019 |
| Value Motivation (B) | | 0.179142 | 0.179151 | | 0.056 | 0.944 | | 0.075 |
| Achievement Motivation (C) | | 0.155750 | 0.155757 | | 0.050 | 0.950 | | 0.105 |
| Knowledge Motivation (D) | | 0.274910 | 0.274922 | | 0.013 | 0.987 | | 0.013 |
| Ability Motivation (E) | | 0.123031 | 0.123037 | | 0.908 | 0.092 | | 0.230 |

*Note*. All probabilities based on randomization tests.

### Table 16 Model 3 Regression Analysis Data

| MODEL FIT | | | | | | | | |
| --- | --- | --- | --- | --- | --- | --- | --- | --- |
| R-square | | Adjusted R-square | | F Value | | | One-Tailed Probability | |
| 0.402 | | 0.325 | | 5.627 | | | 0.029 | |
| REGRESSION COEFFICIENTS | | | | | | | | |
| Independent | Un-stdized Coefficient | | St'dized Coefficient | | Proportion As Large | Proportion As Small | | Proportion As Extreme |
| Intercept | 0.303112 | | 0.000000 | | 1.000 | 0.000 | | 1.000 |
| Gender | 0.090448 | | 0.042528 | | 0.337 | 0.663 | | 0.881 |
| Ages | -0.182237 | | -0.117250 | | 0.801 | 0.199 | | 0.312 |
| Language | 0.055970 | | 0.018404 | | 0.039 | 0.961 | | 0.430 |
| Social Motivation (A) | 0.061567 | | 0.061569 | | 0.312 | 0.688 | | 0.654 |
| Value Motivation (B) | 0.090576 | | 0.090580 | | 0.182 | 0.818 | | 0.371 |
| Achievement Motivation (C) | 0.115016 | | 0.115021 | | 0.146 | 0.854 | | 0.278 |
| Knowledge Motivation (D) | 0.079344 | | 0.079348 | | 0.252 | 0.748 | | 0.493 |
| Ability Motivation (E) | -0.076803 | | -0.076807 | | 0.780 | 0.220 | | 0.431 |
| Achievement Performance (F) | 0.333774 | | 0.333789 | | 0.010 | 0.990 | | 0.019 |
| Adaptability (G) | 0.298206 | | 0.298220 | | 0.017 | 0.983 | | 0.017 |
| Satisfaction (H) | -0.190113 | | -0.190122 | | 0.960 | 0.040 | | 0.076 |

*Note*. All probabilities based on randomization tests.

### Table 17 Model 4 Regression Analysis Data

| MODEL FIT | | | | | | | | |
| --- | --- | --- | --- | --- | --- | --- | --- | --- |
| R-square | | Adjusted R-square | | F Value | | | One-Tailed Probability | |
| 0.538 | | 0.379 | | 3.453 | | | 0.136 | |
| REGRESSION COEFFICIENTS | | | | | | | | |
| Independent | Un-stdized Coefficient | | St'dized Coefficient | | Proportion As Large | Proportion As Small | | Proportion As Extreme |
| Intercept | 0.348147 | | 0.000000 | | 1.000 | 0.000 | | 1.000 |
| Gender | 0.118421 | | 0.055681 | | 0.315 | 0.685 | | 0.755 |
| Ages | -0.201580 | | -0.129696 | | 0.826 | 0.174 | | 0.315 |
| Language | 0.033470 | | 0.011005 | | 0.150 | 0.850 | | 0.657 |
| Social Motivation (A) | 0.103130 | | 0.103134 | | 0.201 | 0.799 | | 0.466 |
| Value Motivation (B) | 0.064468 | | 0.064471 | | 0.287 | 0.713 | | 0.596 |
| Achievement Motivation (C) | 0.191006 | | 0.191015 | | 0.091 | 0.909 | | 0.156 |
| Knowledge Motivation (D) | 0.019837 | | 0.019838 | | 0.391 | 0.609 | | 0.869 |
| Ability Motivation (E) | -0.089029 | | -0.089033 | | 0.822 | 0.178 | | 0.421 |
| Achievement Performance (F) | 0.312062 | | 0.312077 | | 0.055 | 0.945 | | 0.120 |
| Adaptability (G) | 0.289940 | | 0.289953 | | 0.057 | 0.943 | | 0.065 |
| Satisfaction (H) | -0.175860 | | -0.175868 | | 0.906 | 0.094 | | 0.163 |
| A*F | -0.167195 | | -0.227778 | | 0.973 | 0.027 | | 0.110 |
| A*G | 0.173472 | | 0.169016 | | 0.110 | 0.890 | | 0.198 |
| A*H | -0.102737 | | -0.104244 | | 0.850 | 0.150 | | 0.317 |
| B*F | 0.000768 | | 0.000858 | | 0.495 | 0.505 | | 1.000 |
| B*G | -0.014690 | | -0.016961 | | 0.609 | 0.391 | | 0.874 |
| B*H | -0.041404 | | -0.044416 | | 0.652 | 0.348 | | 0.702 |
| C*F | -0.050868 | | -0.062448 | | 0.709 | 0.291 | | 0.555 |
| C*G | 0.088225 | | 0.091726 | | 0.197 | 0.803 | | 0.438 |
| C*H | 0.009947 | | 0.010617 | | 0.387 | 0.613 | | 0.919 |
| D*F | 0.133805 | | 0.142358 | | 0.136 | 0.864 | | 0.260 |
| D*G | -0.080823 | | -0.083455 | | 0.812 | 0.188 | | 0.430 |
| D*H | -0.124233 | | -0.128488 | | 0.876 | 0.124 | | 0.276 |
| E*F | -0.013793 | | -0.014577 | | 0.605 | 0.395 | | 0.935 |
| E*G | 0.017607 | | 0.015570 | | 0.487 | 0.513 | | 0.874 |
| E*H | -0.025999 | | -0.027588 | | 0.594 | 0.406 | | 0.817 |

*Note*. All probabilities based on randomization tests.

### Table 18 Model 5 Regression Analysis Data

| CORRELATION MATRIX | | | | | | | | | |
| --- | --- | --- | --- | --- | --- | --- | --- | --- | --- |
|  | | Gender | | Ages | | Language | | Cognitive Learning Outcomes | |
| Gender | | 1.000 | | 0.072 | | 0.097 | | -0.045 | |
| Ages | | 0.072 | | 1.000 | | -0.135 | | 0.042 | |
| Language | | 0.097 | | -0.135 | | 1.000 | | 0.026 | |
| Non-Cognitive Learning Outcomes | | -0.045 | | 0.042 | | 0.026 | | 1.000 | |
| Determinant | | 0.96511330 | | | | | | | |
| MODEL FIT | | | | | | | | | |
| R-square | | Adjusted R-square | | F Value | | One-Tailed Probability | | | |
| 0.005 | | -0.034 | | 0.183 | | 0.824 | | | |
| REGRESSION COEFFICIENTS | | | | | | | | | |
| Independent | Un-stdized Coefficient | | St'dized Coefficient | | Proportion As Large | | Proportion As Small | | Proportion As Extreme |
| Intercept | -0.292189 | | 0.000000 | | 1.000 | | 0.000 | | 1.000 |
| Gender | -0.111952 | | -0.052639 | | 0.418 | | 0.582 | | 0.935 |
| Ages | 0.078686 | | 0.050626 | | 0.118 | | 0.882 | | 0.335 |
| Language | 0.111769 | | 0.037726 | | 0.438 | | 0.562 | | 0.551 |

*Note*. All probabilities based on randomization tests.

### Table 19 Model 6 Regression Analysis Data

| MODEL FIT | | | | | | | |
| --- | --- | --- | --- | --- | --- | --- | --- |
| R-square | | Adjusted R-square | | F Value | | One-Tailed Probability | |
| 0.162 | | 0.083 | | 2.292 | | 0.063 | |
| REGRESSION COEFFICIENTS | | | | | | | |
| Independent | Un-stdized Coefficient | | St'dized Coefficient | Proportion As Large | Proportion As Small | | Proportion As Extreme |
| Intercept | -0.065275 | | 0.000000 | 1.000 | 0.000 | | 1.000 |
| Gender | -0.307419 | | -0.144547 | 1.000 | 0.000 | | 0.208 |
| Ages | 0.204276 | | 0.131430 | 0.139 | 0.861 | | 0.315 |
| Language | -0.063657 | | -0.020931 | 0.817 | 0.183 | | 0.202 |
| Social Motivation (A) | 0.273044 | | 0.273056 | 0.008 | 0.992 | | 0.008 |
| Value Motivation (B) | 0.059636 | | 0.059639 | 0.252 | 0.748 | | 0.516 |
| Achievement Motivation (C) | -0.190196 | | -0.190204 | 0.993 | 0.007 | | 0.040 |
| Knowledge Motivation (D) | 0.264337 | | 0.264349 | 0.016 | 0.984 | | 0.016 |
| Ability Motivation (E) | 0.028073 | | 0.028075 | 0.404 | 0.596 | | 0.828 |

*Note*. All probabilities based on randomization tests.

### Table 20 Model 7 Regression Analysis Data

| MODEL FIT | | | | | | | | |
| --- | --- | --- | --- | --- | --- | --- | --- | --- |
| R-square | | Adjusted R-square | | F Value | | | One-Tailed Probability | |
| 0.233 | | 0.134 | | 2.535 | | | 0.077 | |
| REGRESSION COEFFICIENTS | | | | | | | | |
| Independent | Un-stdized Coefficient | | St'dized Coefficient | | Proportion As Large | Proportion As Small | | Proportion As Extreme |
| Intercept | -0.163904 | | 0.000000 | | 1.000 | 0.000 | | 1.000 |
| Gender | -0.279499 | | -0.131419 | | 1.000 | 0.000 | | 0.271 |
| Ages | 0.289763 | | 0.186432 | | 0.075 | 0.925 | | 0.105 |
| Language | -0.161744 | | -0.053184 | | 0.995 | 0.005 | | 0.022 |
| Social Motivation (A) | 0.166600 | | 0.166608 | | 0.094 | 0.906 | | 0.165 |
| Value Motivation (B) | 0.017152 | | 0.017153 | | 0.392 | 0.608 | | 0.859 |
| Achievement Motivation (C) | -0.266572 | | -0.266584 | | 1.000 | 0.000 | | 0.027 |
| Knowledge Motivation (D) | 0.160271 | | 0.160279 | | 0.117 | 0.883 | | 0.207 |
| Ability Motivation (E) | -0.004886 | | -0.004886 | | 0.511 | 0.489 | | 0.975 |
| Achievement Performance (F) | 0.108307 | | 0.108312 | | 0.163 | 0.837 | | 0.362 |
| Adaptability (G) | 0.206049 | | 0.206058 | | 0.063 | 0.937 | | 0.082 |
| Satisfaction (H) | 0.228771 | | 0.228781 | | 0.000 | 1.000 | | 0.014 |

*Note*. All probabilities based on randomization tests.

### Table 21 Model 8 Regression Analysis Data

| MODEL FIT | | | | | | | | |
| --- | --- | --- | --- | --- | --- | --- | --- | --- |
| R-square | | Adjusted R-square | | F Value | | | One-Tailed Probability | |
| 0.476 | | 0.295 | | 2.692 | | | 0.150 | |
| REGRESSION COEFFICIENTS | | | | | | | | |
| Independent | Un-stdized Coefficient | | St'dized Coefficient | | Proportion As Large | Proportion As Small | | Proportion As Extreme |
| Intercept | -0.630695 | | 0.000000 | | 1.000 | 0.000 | | 1.000 |
| Gender | -0.228964 | | -0.107658 | | 0.797 | 0.203 | | 0.463 |
| Ages | 0.369332 | | 0.237626 | | 0.021 | 0.979 | | 0.044 |
| Language | -0.079000 | | -0.025976 | | 0.735 | 0.265 | | 0.328 |
| Social Motivation (A) | 0.051315 | | 0.051317 | | 0.344 | 0.656 | | 0.733 |
| Value Motivation (B) | 0.037419 | | 0.037421 | | 0.379 | 0.621 | | 0.779 |
| Achievement Motivation (C) | -0.284408 | | -0.284421 | | 0.992 | 0.008 | | 0.056 |
| Knowledge Motivation (D) | 0.150793 | | 0.150800 | | 0.121 | 0.879 | | 0.233 |
| Ability Motivation (E) | -0.008082 | | -0.008082 | | 0.537 | 0.463 | | 0.937 |
| Achievement Performance (F) | 0.211270 | | 0.211279 | | 0.111 | 0.889 | | 0.220 |
| Adaptability (G) | 0.261604 | | 0.261616 | | 0.065 | 0.935 | | 0.077 |
| Satisfaction (H) | 0.154114 | | 0.154121 | | 0.105 | 0.895 | | 0.198 |
| A*F | 0.254437 | | 0.346631 | | 0.033 | 0.967 | | 0.033 |
| A*G | -0.223334 | | -0.217596 | | 0.960 | 0.040 | | 0.110 |
| A*H | 0.140077 | | 0.142131 | | 0.134 | 0.866 | | 0.233 |
| B*F | -0.223136 | | -0.249417 | | 0.984 | 0.016 | | 0.060 |
| B*G | 0.079665 | | 0.091981 | | 0.220 | 0.780 | | 0.389 |
| B*H | -0.051466 | | -0.055210 | | 0.694 | 0.306 | | 0.655 |
| C*F | 0.077718 | | 0.095411 | | 0.186 | 0.814 | | 0.382 |
| C*G | -0.035206 | | -0.036604 | | 0.547 | 0.453 | | 0.725 |
| C*H | -0.096085 | | -0.102562 | | 0.808 | 0.192 | | 0.370 |
| D*F | -0.015490 | | -0.016480 | | 0.491 | 0.509 | | 0.885 |
| D*G | 0.201344 | | 0.207900 | | 0.057 | 0.943 | | 0.088 |
| D*H | -0.010449 | | -0.010806 | | 0.595 | 0.405 | | 0.900 |
| E*F | 0.185971 | | 0.196541 | | 0.099 | 0.901 | | 0.187 |
| E*G | 0.047335 | | 0.041858 | | 0.379 | 0.621 | | 0.674 |
| E*H | 0.063889 | | 0.067792 | | 0.263 | 0.737 | | 0.512 |

*Note*. All probabilities based on randomization tests.
